# Family‐based interventions to increase physical activity in children: a systematic review, meta‐analysis and realist synthesis

**DOI:** 10.1111/obr.12362

**Published:** 2016-01-12

**Authors:** H. E. Brown, A. J. Atkin, J. Panter, G. Wong, M. J. M. Chinapaw, E. M. F. van Sluijs

**Affiliations:** ^1^ MRC Epidemiology Unit and UKCRC Centre for Diet and Activity Research (CEDAR)University of Cambridge School of Clinical MedicineCambridgeUnited Kingdom; ^2^ Nuffield Department of Primary Care Health SciencesUniversity of OxfordOxfordUnited Kingdom; ^3^ Department of Public and Occupational Health, EMGO Institute for Health and Care ResearchVU University Medical CentreAmsterdamThe Netherlands

**Keywords:** Family, physical activity, interventions

## Abstract

**Objective:**

Family‐based interventions represent a potentially valuable route to increasing child physical activity (PA) in children. A dual meta‐analysis and realist synthesis approach examined existing interventions to assist those developing programmes to encourage uptake and maintenance of PA in children.

**Design:**

Studies were screened for inclusion based on including participants aged 5–12 years, having a substantive aim of increasing PA by engaging the family and reporting on PA outcome. Duplicate data extraction and quality assessment were conducted. Meta‐analysis was conducted in STATA. Realist synthesis included theory development and evidence mapping.

**Results:**

Forty‐seven studies were included, of which three received a ‘strong’ quality rating, 21 ‘moderate’ and 23 ‘weak’. The meta‐analysis (19 studies) demonstrated a significant small effect in favour of the experimental group (standardized mean difference: 0.41; 95%CI 0.15–0.67). Sensitivity analysis, removing one outlier, reduced this to 0.29 (95%CI 0.14–0.45). Realist synthesis (28 studies) provided insight into intervention context (particularly, family constraints, ethnicity and parental motivation), and strategies to change PA (notably, goal‐setting and reinforcement combined).

**Conclusion:**

This review provides key recommendations to inform policy makers and other practitioners in developing evidence‐based interventions aimed at engaging the family to increase PA in children, and identifies avenues for future research.

## Background

The positive impact of physical activity on health is well established. In children, physical activity is favourably associated with cardiovascular risk factors 1, 2, 3, anthropometric indicators (particularly body composition, waist circumference and fat mass) 2, 4, 5 and bone health 6. Regular engagement in physical activity is also beneficial for young people's mental health and self‐esteem, and has been suggested to support improved cognitive performance and scholastic achievement 7, 8, 9. Despite these known benefits, data from several countries suggest that the majority of children are insufficiently active to confer health benefit 10, 11, and that levels of physical activity decline substantially throughout childhood and into adolescence 12, 13. Intervening prior to the steep decline observed in adolescence may be important to maintain adequate physical activity levels 14. The development of interventions to promote and maintain children's activity levels is therefore a public health priority.

Physical activity promotion is predominantly conducted in schools, but the impact of school‐based interventions on overall physical activity has been questioned 15, 16. Recent publications state that *without* the involvement of family members, it is unlikely that long‐term change in children's physical activity levels is possible 17, 18. Parental support, such as provision of transport, co‐participation or encouragement, has been consistently and positively associated with youth physical activity, particularly in children 19, 20, and the addition of a family component (e.g. parent education) to school‐based interventions has shown to be efficacious 16.

Despite their potential, little is known about how best to engage families in physical activity promotion 21, 22, 23. Conclusions from reviews of extant trials have been limited by heterogeneity in target populations, and variability in study design and outcome measures 16, 21, 24, 25. Considering the complexity of the existing evidence, a multi‐faceted approach to synthesis is required, to enable enhancement of the conclusions that could be drawn from any single method. A dual approach, combining meta‐analytic and realist synthesis techniques, augments our understanding by not only identifying the effectiveness of interventions to date, but also the key characteristics of effective interventions and the contexts in which they operate. By combining two evidence synthesis methodologies, this review will provide the most comprehensive examination to date of existing family‐based interventions and with that, inform doctors, policy makers and other practitioners in developing and applying physical activity promotion programmes 14. Meta‐analysis was applied to examine overall intervention effect, and a realist synthesis approach employed to explore ‘what works for whom, under what circumstances, how, and why?’^26^. Using this dual approach, the objective of this study was to review existing intervention studies which explicitly engage the family to increase physical activity in children.

## Methods

The review protocol was registered with the International Prospective Register for Systematic Reviews (PROSPERO) CRD42013005780, and we refer to the published protocol for full details of the review procedures 14. In brief, peer‐reviewed experimental studies (of any design) published up to and including September 2015 from a literature search of PubMed, Web of Knowledge, Scopus, Ovid MEDLINE and PsycInfo, and screened in duplicate (HEB/AA/EvS) using the following inclusion criteria (i) including ‘healthy’ participants aged 5–12 years, (ii) having a substantive intervention aim of increasing physical activity, by actively engaging the family (i.e. intervention effect could be attributed to large family component) and (iii) reporting on any physical activity outcome assessed using a measure (i.e. by self‐ or proxy‐report questionnaire or diary, pedometer, accelerometer, inclinometer or heart rate monitor). All study designs were included. The search strategy and terms used are reported in Supporting Information 1. Where available, supplementary data cited in each paper were accessed (for example, adding process evaluation or protocol papers). Duplicate data extraction and quality assessment were conducted (HEB/MCAP) using a specially designed pro‐forma and the Effective Public Health Practice Project (EPHPP) Quality Assessment Tool, respectively. Conflicts regarding all screening and review procedures were resolved by a third reviewer.

### Meta‐analysis

Meta‐analysis was conducted in STATA (Version 13; StataCorp. 2013, TX: StataCorp LP). It only included those studies reporting sufficient data on the effect of the intervention on physical activity to calculate a standardized effect size (i.e. mean, standard deviation and sample size, for both control and intervention groups). Standardized mean differences were calculated (using the mean and standard deviation of the treatment group and control group for each study): Hedge's g = *M*_1_ − *M*_2_ / SD*_pooled_ (where SD_pooled_ = √[(n_1_ − 1)SD_1_^2^ + (n_2_ − 1) SD_2_^2^ / n_1_ + n_2_ − 2]), and combined using a random‐effects model to derive an overall summary effect estimate (and 95% CI). Size of effect was broadly categorized using existing criteria (≤0.2: small, ≤0.5: moderate and ≤0.8: large 27). Data was extracted by HEB and checked for accuracy by a second reviewer (KC). When the required data were not available, study authors were contacted with requests for relevant information (this represents a minor adjustment from the published protocol 14, and was conducted to ensure all possible studies were included for meta‐analysis). The type of data extracted differed according to the data reported in each paper (such as model statistics, coefficients, between‐group mean differences and within‐group means). Corresponding measures of precision, including standard deviations, standard errors, 95% confidence intervals (CIs) and number of participants analysed, were also extracted. There were no included studies stratified by sex; where studies stratified results by several intervention types (e.g. family only, family + community, community only 28), the effect size most relevant to the review objective was selected via consensus between two authors (HEB and EvS). Heterogeneity between studies was quantified using the I^2^ statistic.

### Realist synthesis

A review based on realist principles was conducted using the RAMESES quality and publication guidelines 26, 29. The primary objective of the realist synthesis was to explain the outcome patterns reported within the wider systematic review. An initial programme theory (i.e. a hypothesized causal model) was developed with input from all authors through a consensus process facilitated by GW. This programme theory described the hypothesized context and mechanisms necessary to elicit a specified outcome (such as increased weekend physical activity in children), and was used as a template against which included studies were later mapped.

Data (e.g. verbatim sections of text) from each of the included studies and any relevant sibling papers was coded using nVivo analysis software (coded first by one author (HEB), and then all interpretations were checked by a second author (JP)). Where available, information on full or partial Context–Mechanism–Outcome (CMO) configurations was extracted for individual studies (i.e. we sought out data that would explain what caused an Outcome [Mechanism] and under which Contexts). Data were included in the realist synthesis based on the principles of ‘relevance’ (whether it can contribute to theory building) and ‘rigour’ (whether the methods used to generate the relevant data are credible and trustworthy) 26, 29. Text pertaining to evidence, or hypotheses as to how specific elements of the intervention (might have) worked, was considered; information pertaining to theory description were retained for information purposes only (i.e. to provide relevant background). CMO configurations were then mapped on the initial programme theory to form individual diagrams for each of the 47 studies, which were further reviewed and agreed upon by three authors (HEB, EvS and JP). The programme theory was further developed throughout this process, adjusting dynamically to reflect the evidence from included studies. Finally, in a consensus meeting, patterns in outcomes (termed demi‐regularities 26, 29) across all study diagrams were configured into a final realist programme theory (a refined and evidence‐supported version of the initial programme theory), with the aim of identifying (where possible) which *context* triggers which *mechanism*, and in turn might elicit which *outcome*. Where appropriate, theories cited in the included interventions were studied, to provide additional insight into possible or missing mechanisms.

## Results

Given the range of evaluative techniques applied to studies in this review, findings are presented in four sections; the first two describing the study characteristics and quality, the latter describing the results of the meta‐analysis and realist synthesis.

### Study characteristics

**Figure 1:**
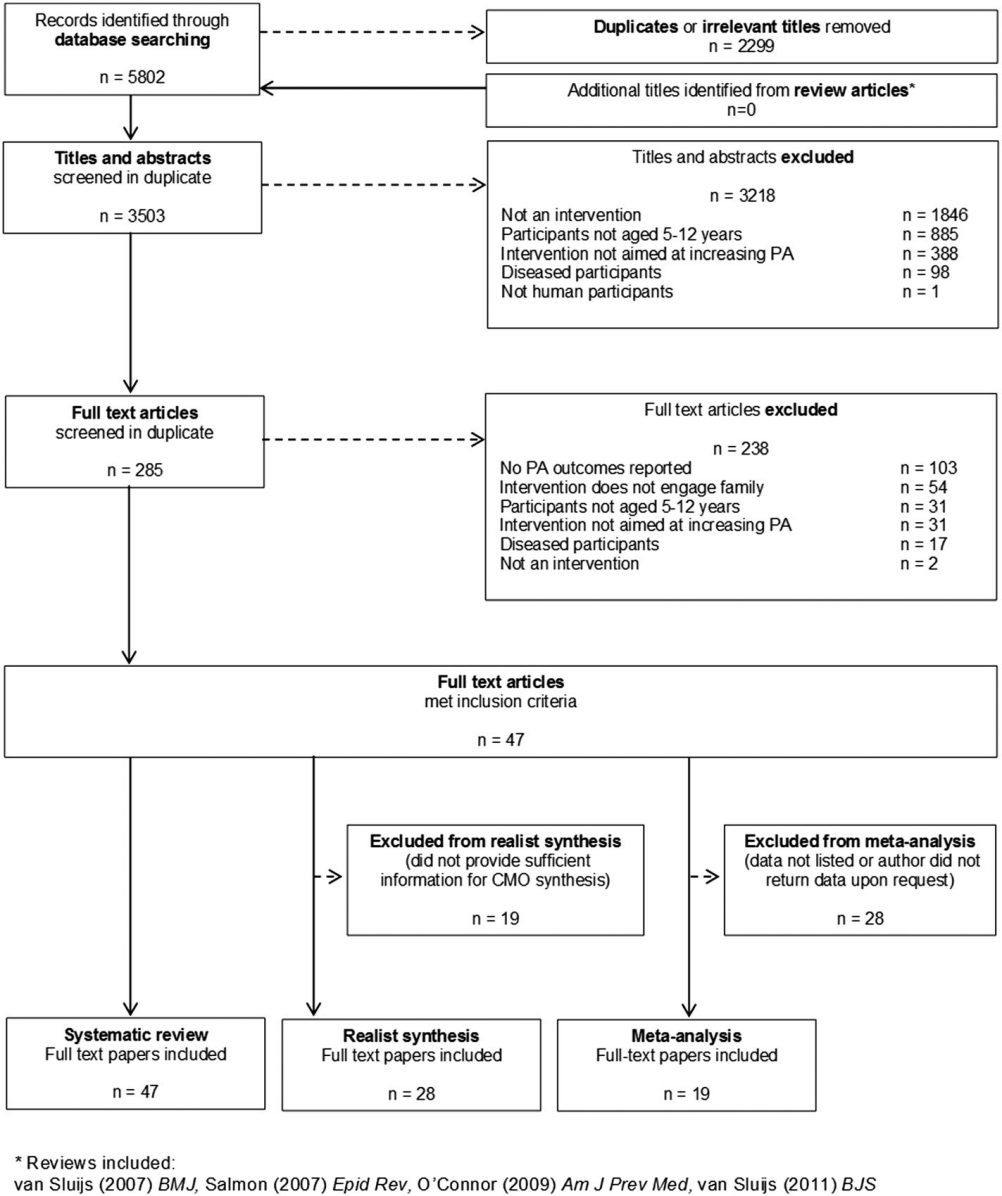
Evidence search and exclusion process.

Forty‐seven interventions met inclusion criteria 28, 30, 31, 32, 33, 34, 35, 36, 37, 38, 39, 40, 41, 42, 43, 44, 45, 46, 47, 48, 49, 50, 51, 52, 53, 54, 55, 56, 57, 58, 59, 60, 61, 62, 63, 64, 65, 66, 67, 68, 69, 70, 71, 72, 73, 74 (Fig. 1), of which 31 (66%) demonstrated a significant positive effect on physical activity. Table 1 provides an overview of included studies, grouped by study characteristics and demonstrating intervention effect. Further details on each of the studies, including a description of the intervention and the evaluation used to assess effectiveness, are provided in Supplementary Table 1.

**Table 1:** Overview of characteristics of 47 studies included in systematic review of family‐based physical activity promotion

	Proportion	Citations	Favoured intervention #
**Study design**			
Randomized controlled trials (RCT)/cluster RCT	57%	1,2,3,4,5,6,7,8,9,10,11,12,13,14,15,16,17,18,19,20,21,22,23,24,25,26,27	59%
Comparison trials	26%	28,29,30,31,32,33,34,35,36,37,38,39	67%
Pilot studies or feasibility trials of any design	17%	40,41,42,43,44,45,46,47	88%
**Year of publication**			
2014–2015	6%	14,23,39,46	75%
2012–2013	36%	1,4,5,10,21,24,25,26,28,31,35,40,41,42,44,45,47	71%
2010–2011	26%	6,7,8,9,11,27,30,33,36,37,38,43	67%
1983–2009	32%	2,3,12,13,15,16,17,18,19,20,22,29,32,34	57%
**Study location**			
USA	59%	1,2,6,7,11,13,15,16,17,18,19,21,22,23,29,30,31,32,34,35,36,37,39,40,41,44,45,46	68%
UK	15%	8,12,26,28,38,42,47	58%
Australia	11%	14,20,25,27,33	20%
Other locations +	15%	3,4,5,9,10,24,43	100%
**Sample size (‘target’ child)**			
<60 participants	45%	2,6,8,12,20,21,25,26,27,29,30,32,36,37,39,40,41,42, 44,45,46	57%
60–200 participants	32%	3,7,9,13,14,16,18,22,31,33,34,35,38,43,45	73%
≥200 participants	23%	1,4,5,10,11,15,19,23,24,28,47	72%
**Age of target child**			
5–8 years	15%	1,20,23,24,42,43,47	71%
8–11 years	70%	3,4,5,7,8,9,10,11,12,14,17,19,21,22,25,26,27,28,29,30,31,32,33,34,35,36,37,38,39,41,44,45,46	60%
≥11 years	13%	6,13,15,16,18,40	50%
Not reported	2%	2	0%
**Sex of target child**			
Mixed sex	83%	1,2,3,4,5,6,7,8,9,10,11,13,14,15,16,18,19,20,21,22,23,24,25,26,27,28,30,31,32,33,34,35,38,39,40,41,42,43, 44,47	63%
Girls only	15%	12,17,29,36,37,45,46	86%
Not reported	2%	42	100%
**Weight status of target child**			
Majority healthy weight	43%	1,3,4,5,6,7,10,11,12,14,18,23,24,26,29,31,32,37,38,47	80%
Majority overweight or obese	36%	1,8,18,19,20,21,27,28,30,33,34,35,37,39,40,41,44	59%
Not reported	21%	2,9,13,15,22,25,41,42,43,46	50%
**Follow‐up (post‐intervention) periods reported**			
Short term: up to 6 months	51%	9,10,13,15,16,17,18,19,20,21,22,25,26,27,29,31,36,39,41,42,43,44,46,47	58%
Medium term: 6–12 months	19%	3,4,6,7,8,14,17,21,32	89%
Long term: 12 months or longer	30%	1,4,8,10,13,15,19,21,23,24,27,30,33,34	79%
**Physical activity measure** *			
Subjective (e.g. questionnaire, recall diary, interview)	53%	1,3,4,5,11,13,15,16,19,20,21,22,27,28,30,32,34,35,38,40,41,43,44,46,47	68%
Objective (e.g. pedo/accelerometry (Actical, ActiGraph or Caltrac devices), observation)	46%	2,6,7,8,9,10,12,14,17,18,23,24,25,26,29,31,33,36,37, 39,42,45	64%
**Physical activity outcome reported**			
Accelerometer‐derived MVPA or counts/min	34%	6,7,8,17,18,21,23,24,26,29,31,33,36,37,42,45	63%
Pedometer‐derived step count	15%	9,10,12,14,25,39,41	71%
Self‐reported PA frequency (>60 min)	45%	1,3,11,13,15,16,19,20,22,27,28,30,32,34,35,38,40,43,44,46,47	67%
Self‐reported sport, dance, PE or outdoor play participation or direct observation	6%	2,4,5	67%
**Theoretical grounding**			
No theory identified	43%	5,8,10,12,17,18,19,20,21,22,26,27,28,30,31,38,40,41,44,47	60%
Theory‐based	57%	1,2,3,4,6,7,9,11,13,14,16,23,24,25,29,32,33,34,35,36,37,39,42,43,45,46	74%
**Intervention duration**			
≤1 month	17%	12,20,22,32,34,36,41,43	63%
1–2 months	23%	4,6,7,9,13,16,25,27,40,42,46	64%
>2 to 3 months	34%	14,18,19,21,26,28,29,30,31,33,35,37,38,39,44,47	69%
>3 months	26%	1,2,3,5,8,10,11,15,17,23,24,45	67%
**Intervention deliverer**			
Community leaders	19%	1,3,13,15,22,29,30,44,45	67%
Medical or healthcare staff	23%	4,5,7,8,20,21,23,28,36,37,47	55%
Research team	19%	9,14,16,25,26,32,34,42,46	67%
Remote delivery (online or mail)	15%	6,24,31,39,40,41,43	71%
Teaching staff (specializing in Physical Education)	2%	33	0%
Not reported	21%	2,10,11,12,17,18,19,30,35,38	90%
**Intervention strategy applied** †			
Education	89%	1,2,3,4,5,6,7,8,9,10,11,12,13,14,15,16,17,18,19,20,21,22,23,24,26,27,28,29,30,31,32,33,34,35,36,37,38,40, 41,42,43,44,45,46,47	67%
Goal‐setting	40%	1,2,3,5,6,12,13,15,16,18,19,23,25,30,31,39,41,45,46	53%
Reinforcement of positive health behaviours	17%	5,9,10,11,12,13,14,15,19,20,25,26,33,39,41,45	56%
Role modelling	17%	3,12,13,14,20,23,34,39	63%
**Intervention focus**			
PA only	21%	1,10,12,17,22,24,26,31,39,43	70%
Included other behaviour (e.g. diet, screen time)	79%	1,2,3,4,5,6,7,8,9,11,13,14,15,16,18,23,25,27,28,29,30,36,37,38,41,42	62%

# Corresponds with a significant, positive change in outcome (see Supplementary Table 1 for full details).

+ Singapore, Mexico, Italy, New Zealand, Canada and Germany.

* Some studies employed both subjective and objective methods to measure physical activity.

† Some studies employed more than one intervention strategy.

Just over half of the included interventions were evaluated using a randomized controlled trial (RCT) or cluster RCT (*n* = 27, 57%), and 8 (17%) were pilot or feasibility studies (of which 88% demonstrated positive results). Most studies were published after 2010 (68%), offering evidence of the contemporary nature of family‐based physical activity work, and alluding to the growing momentum of research in this setting. The majority of interventions were conducted and evaluated in the USA, but other study locations were included. Almost half of the studies included less than 60 participants and most (70%) included families with children aged between 8 and 11 years. Single‐sex interventions were uncommon; no studies focused on boys only, and only seven studies (15%) focussed specifically on girls (of which 86% reported a favourable effect). Studies targeting both sexes had broadly equal numbers of boys and girls. Studies including a majority of healthy weight participants appeared more effective than those reporting a high proportion of overweight or obese participants (80% compared to 59%, respectively). Approximately 85% of studies included short term post‐intervention follow‐up (≤3 months, *n* = 39), with 30% presenting results of a long term follow‐up (≥12 months, *n* = 14). Physical activity was assessed using subjective methods (questionnaires, recall diaries and interviews) in 25 (53%) studies, whilst objective assessment (pedometry, accelerometry and direct observation) featured in 22 studies (46%). Frequency of physical activity was commonly assessed using self‐report methods (*n* = 21, of which 67% reported a favourable effect), whilst objective methods were used to record time spent in moderate‐vigorous physical activity or accelerometer counts per minute (*n* = 16, 63% favourable), and step counts (*n* = 7, 71% favourable). Overall, evaluations based on self‐report measures were no more likely to report a positive intervention effect (68%, compared to 64% of those using objective measures).

A small majority of studies (57%) cited some theoretical grounding for their intervention (e.g. Social Cognitive Theory or Social Learning Theory). Theory‐based interventions appeared to be marginally more effective than those not referencing a particular theory (74% of those citing some theoretical background, and 60% of those citing none, were effective). Intervention duration ranged from 8 days to 12 months. Interventions were delivered by a variety of facilitators; community leaders (often selected for their cultural connection to participants), medical or healthcare staff, members of the research team or teaching staff. Interventions delivered by medical or healthcare staff appeared least effective. Seven studies evaluated the effect of remote delivery, of which 5 (71%) were effective. Education was provided in almost all interventions; other frequently applied intervention strategies included goal‐setting, reinforcement of positive health behaviours and role modelling.

### Study quality

Supplementary Table 2 presents the results of the individual item and overall scores of the quality assessment. ‘Study design’ was the item upon which most studies (72%) were deemed ‘strong’, followed by accounting for confounders (57%), withdrawal and drop‐out (57%) and data collection methods (43%). Issues of selection bias (e.g. sample representativeness of target population), and participant blinding were inadequately addressed; only eight and four studies respectively received a strong rating for each of these criteria. Overall, only three studies received a ‘strong’ rating (6%), 21 were rated ‘moderate’ (45%) and the remaining 23 were rated as ‘weak’ (49%).

### Meta‐analysis

**Figure 2:**
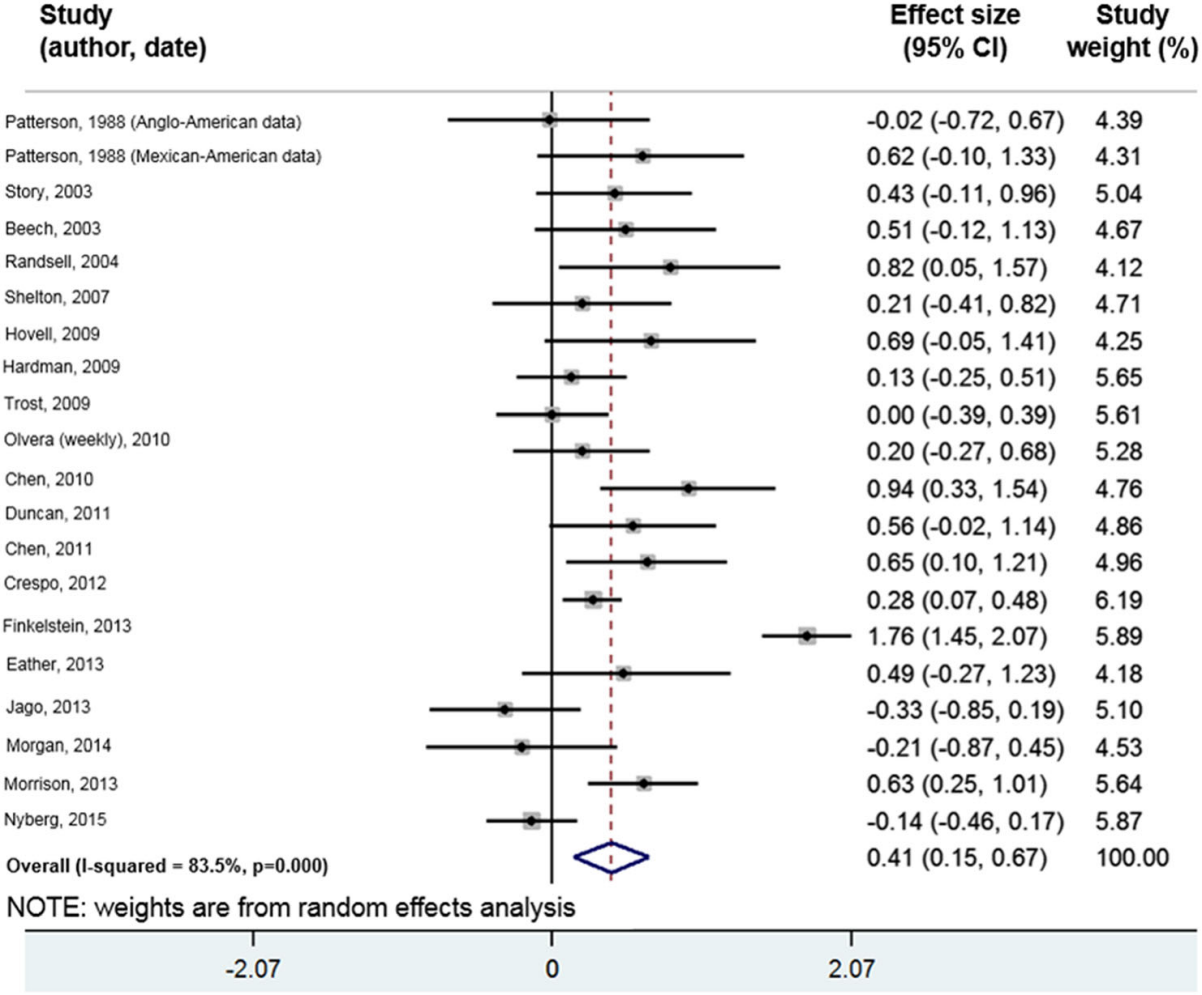
Effect on child physical activity from random effects meta‐analysis of eligible studies.

Nineteen studies, one of strong 74, ten of moderate 30, 35, 41, 47, 53, 58, 67, 68, 69, 70 and eight of weak methodological quality 28, 39, 45, 50, 51, 56, 63, 65 provided sufficient data to be included in the meta‐analysis. Of these, 15 were included based on published data, four on author‐provided data. Other studies reported only graphical information, or did not provide sufficient data to calculate a post‐test mean and standard deviation for each group and were therefore deemed unsuitable for inclusion (these presented, for example, change in pre‐post scores between groups, geometric mean and interquartile range, ‘difference score’). The pooled analysis (including 715 control and 863 intervention participants) showed a statistically significant effect in favour of the intervention groups (standardized mean difference 0.41; 95% CI 0.15–0.67; *p* < 0.000) (Fig. 2); this is deemed a small effect size 27. The *I^2^* value was 83.5%, indicating high heterogeneity 75. Given the weight and magnitude of effect demonstrated by one study 47, sensitivity analyses were conducted to assess the impact of removing this study from the meta‐analysis. In this moderate‐quality study, families received educational materials on the benefits of physical activity, and information about available weekend outdoor activities and pedometer step targets. When this study was removed, the effect was reduced (standardized mean difference 0.29, 95% CI 0.14, 0.45), and the heterogeneity decreased to 45.6%. No sub‐group analyses for type of physical activity measure or study quality were conducted because of lack of heterogeneity (only four studies used a self‐report measure, and only one received a ‘strong’ quality rating).

### Realist synthesis

**Figure 3:**
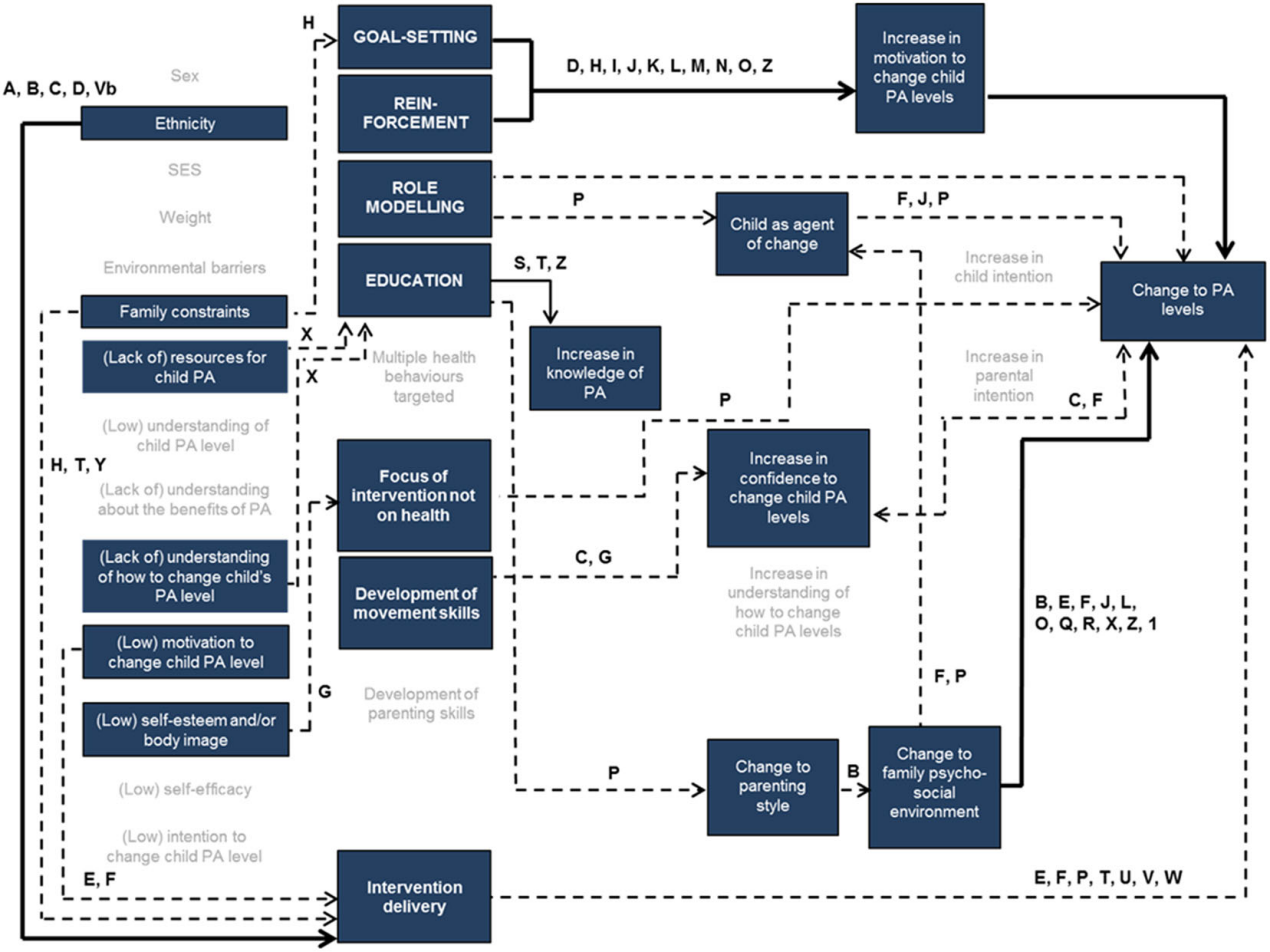
Final programme theory for family‐based physical activity interventions. (1.) Solid arrows indicate configuration between context, mechanisms and outcomes evidenced by the included studies, dashed arrows depict those configurations that were hypothesized but not evidenced. (2.) Arrows are labelled with the studies that have informed them: **A**: ABC; **B**: Aventuras Para Ninos; **C**: Reach Out; **D**: SHARE AP Action; **E**: Family Connections; **F**: Healthy Choices; **G**: HIKCUPS; **H**: *Rhodes*; **I**: Growing Healthy Families; **J**: Healthy Homework; **K**: TEAM; **L**: A Family Affair; **M**: *Hovell*; **N**: *Finkelstein*; **O**: *Centis*; **P**: Healthy Dads, Healthy Kids; **Q**: ABC (internet); **R**: One Body One Life; **S**: GEMS Memphis; **T**: *Chen*; **U**: Triple P; **V**: MEND 7–13; s**Vb**: MEND 5–7; **W**: Fit for Health; **X**: *Arredondo*; **Y**: *De Bock*; **Z**: C‐PET; **1**: Fit4Fun. (3.) Grey text indicates those items that were hypothesized (during the initial programme theory development phase) to be of interest, but were not supported by evidence from the included studies.

**Figure 4:**
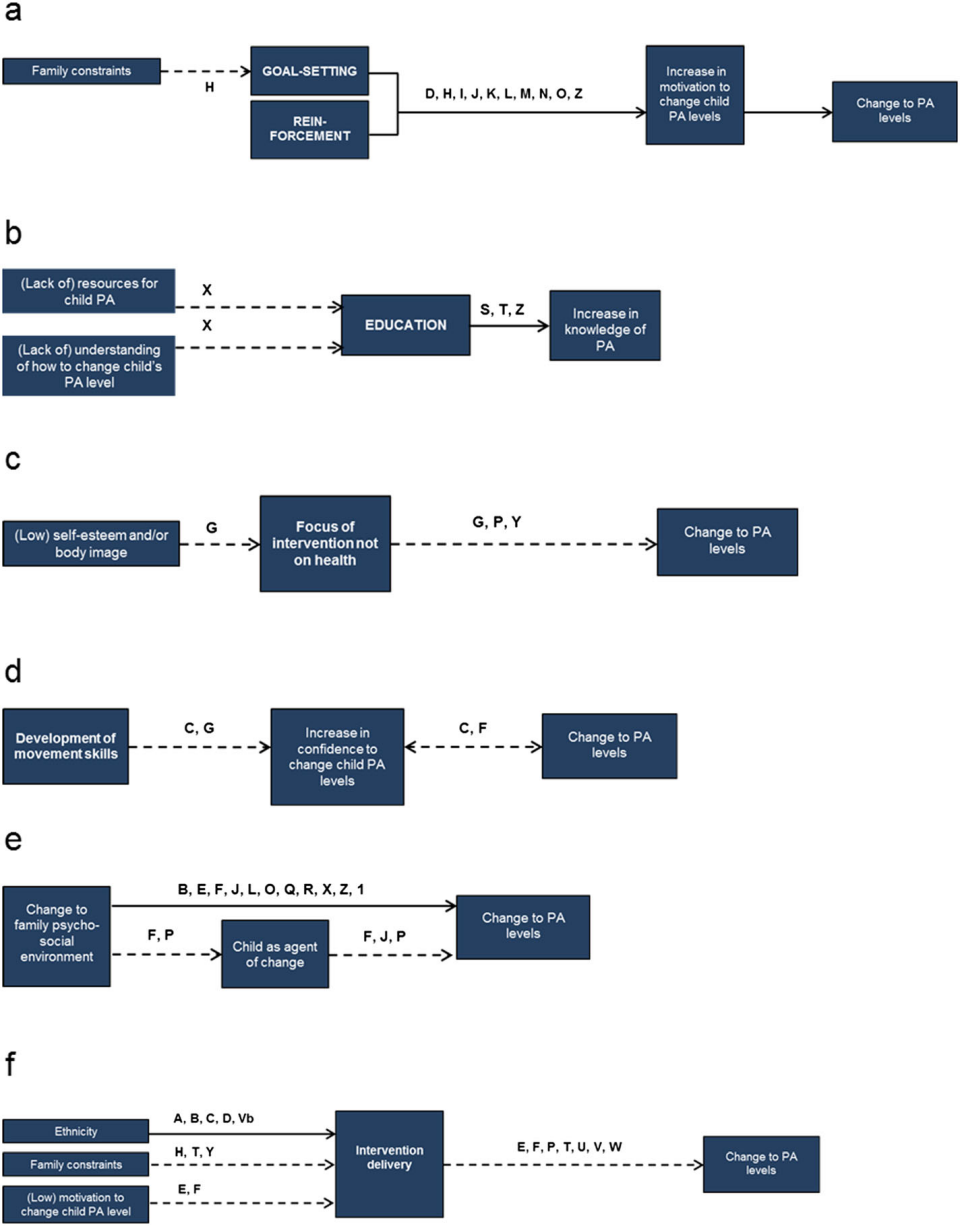
Overview of main patterns identified in realist synthesis of family‐based physical activity interventions. (1.) Solid arrows indicate configuration between context, mechanisms and outcomes evidenced by the included studies; dashed arrows depict those configurations that were hypothesized but not evidenced. (2.) Arrows are labelled with the studies that have informed them: **A**: ABC; **B**: Aventuras Para Ninos; **C**: Reach Out; **D**: SHARE AP Action; **E**: Family Connections; **F**: Healthy Choices; **G**: HIKCUPS; **H**: *Rhodes*; **I**: Growing Healthy Families; **J**: Healthy Homework; **K**: TEAM; **L**: A Family Affair; **M**: *Hovell*; **N**: *Finkelstein*; **O**: *Centis*; **P**: Healthy Dads, Healthy Kids; **Q**: ABC (internet); **R**: One Body One Life; **S**: GEMS Memphis; **T**: *Chen*; **U**: Triple P; **V**: MEND 7–13; **Vb**: MEND 5–7; **W**: Fit for Health; **X**: *Arredondo*; **Y**: *De Bock*; **Z**: C‐PET; **1**: Fit4Fun. (3.) Grey text indicates those items that were hypothesized (during the initial programme theory development phase) to be of interest, but were not supported by evidence from the included studies.

Although all 47 studies were potentially eligible for inclusion in the realist synthesis, only 28 provided sufficient information to describe outcome patterns (defined as being of adequate ‘relevance’ and ‘rigour’ 26, 29) and hence contributed to the realist synthesis 31, 33, 34, 35, 36, 38, 39, 40, 41, 44, 45, 46, 47, 49, 51, 52, 59, 60, 61, 62, 64, 65, 66, 68, 69, 70, 74, 76, 77. These studies displayed great variation in the context within which interventions were conducted, the intervention strategies employed and the (implicit) mechanisms targeted. Despite this heterogeneity, the realist synthesis enabled extraction of demi‐regularities (outcome patterns). The final programme theory demonstrates these patterns (see Fig. 3), illustrating configurations supported by evidence (or hypothesized based on evaluations). Specific configurations of interest are discussed below (see Fig. 4).

In the context of family constraints (such as time or scheduling difficulties), a combination of goal‐setting and reinforcement intervention strategies was effective in changing physical activity behaviour, through the mechanism of increased motivation (see Fig. 4a). Behaviour change theories, such as the Theories of Reasoned Action and Planned Behaviour 78, 79, 80, and the Health Action Process Approach 81, suggest that goal setting behaviours (e.g. thinking through realistic and/or preferred outcomes) increase motivation and promote intention formation. For example, effective family‐based goal‐setting interventions included the study conducted by Rhodes and colleagues 59, which focused on family planning for physical activity. Families were encouraged to plan for ‘when’, ‘where’, ‘how’ and ‘what’ physical activity they would undertake using a provided family calendar and workbook. The authors hypothesized that common barriers (such as perceived limited time available for physical activity because of work or domestic duties 82) may be overcome by increasing parental motivation (Study H, Fig. 4a). Goal setting may provide busy parents with the additional impetus needed to prioritize their child's physical activity above other competing demands. In addition, parents in one pedometer‐based intervention where weekly telephone calls were provided to reflect on progress and encourage greater engagement in changing behaviour were reported to appreciate the systematic reinforcement they received 38. Authors hypothesized that the praise provided during telephone calls encouraged parents to change their physical activity behaviour (and that of their children). This observation is congruent with Self‐Regulation Theory, which suggests that motivation may be increased through observing discrepancies between actual and desired behaviour, a process in which self‐monitoring plays an important role 83.

Our findings suggest that providing education is an effective intervention for changing physical activity knowledge, particularly in the context of a lack of understanding of how to change child physical activity behaviour, and where resources for child physical activity are inadequate (see Fig. 4b). Focus groups, conducted by Arredondo and colleagues to inform intervention development, highlighted parent's lack of knowledge and access to resources 73. The resulting intervention provided weekly information sessions designed to educate mothers and daughters on healthy behaviours. However, this and other studies confirmed that education alone is unlikely to change behaviour 35, 41 (Study X, Fig. 4b). Parents in the *C‐PET* intervention suggested that providing health information *in combination with* reinforcement (such as a reward chart to recognize increased walking) was more effective 70 (Study Z, Fig. 4b). Future efforts should therefore focus on providing feedback or facilitating self‐monitoring (particularly to increase physical activity awareness) to enhance the effect of education.

Most interventions included in this review targeted more than one health behaviour (e.g. physical activity *and* diet), but we did not identify evidence to either support nor refute the effectiveness of this strategy (as compared to targeting physical activity only). However, focusing an intervention on something *other than* the health benefits of physical activity or weight loss appeared to be an effective mechanism for changes in physical activity (see Fig. 4c). The ‘Healthy Dads Healthy Kids’ intervention was marketed as an opportunity for fathers to spend quality time with their children 69. The programme successfully used physical activity as a medium to engage fathers in play with their children, rather than as an explicit strategy for weight loss (Study P, Fig. 4c). Similarly, a parent‐focused intervention that facilitated shared ideas on active transport, ‘lifestyle’ physical activity (e.g. gardening club), promotion of outdoor activities (e.g. forest trips) and reducing obesogenic traditions (e.g. healthier birthday parties), suggested that the character of the intervention as ‘fun’ and ‘childlike’, rather than ‘good for health’, may have contributed to its efficacy 76 (Study Y, Fig. 4c). Other evidence suggested that this strategy may be particularly useful in the context of those with low self‐esteem or poor body image. The ‘HIKCUPS’ intervention did not focus on weight loss (despite being aimed at obese children), but instead focussed on improvements in motor skill proficiency and perceived physical activity competence as alternative benefits 42 (Study G, Fig. 4c).

The focus on the development of movement skills in *HIKCUPS* 42 is hypothesized to have impacted on the children's confidence to change their own physical activity behaviour (see Fig. 4d). Children's confidence (identified as both a mechanism and an intermediate outcome) is further suggested to have a bi‐directional relationship with physical activity, as evidenced by the *Reach Out* and *Healthy Choices* interventions 36, 52. *Healthy Choices* focused on building cognitive and behavioural skills to support physical activity, with learning experiences designed to improve perceived competence in a supportive environment 52 (Study F, Fig. 4d). Whilst it was not possible to clearly identify the underpinning mechanism(s) for the relationship between confidence and behaviour change, it is consistent with the Social Cognitive Theory, which asserts that behaviour is directly influenced by self‐efficacy (people's belief in their ability to perform a specific action that is required to attain an expected outcome) 83.

Consistent support was found for changes to the family psycho‐social environment as a target for intervention for positive changes in physical activity behaviour, either directly 28, 34, 38, 39, 45, 46, 66, or via the child as the agent of change 64, 84 (see Fig. 4e). The *C‐PET* intervention targeted modifications of the family environment, and the importance of family support for physical activity was emphasized throughout 70 (Study Z, Fig. 4e). Authors describing *A Family Affair* report that an improved daughter–mother relationship led to greater support for a healthier lifestyle, namely via co‐participation in physical activity 34. This intervention included family celebrations to further emphasize the role of the mother–daughter relationship (Study L, Fig. 4e). The *One Body One Life* intervention asserted that physical activity choices made by family members will positively affect the home environment, and may subsequently make it more conducive to behaviour change 66 (Study R, Fig. 4e). Family Systems Theory supports this notion; it suggests that an individual's functioning is integrally tied to the functioning of other family members, which may lead to complementary patterns of behaviour 85. A virtuous cycle (in which complex chains of events reinforce themselves through a feedback loop) may occur when one family member becomes more physically active, prompting others to follow and engage in activity themselves. However, whereas this may impact on increase family co‐participation in physical activity, the effect on child physical activity may be limited because of displacement (e.g. children replacing physical activity after school with family dog walks), as suggested in C‐PET 70.

It is also important to note that, conversely, a lack of family support may restrict healthy behaviour change. The process evaluation of the *Fit4Fun* intervention highlighted that children commented that their ‘parents did not really encourage [me] to do the physical activities’, and hypothesized that poor family engagement may have contributed to the only modest improvements in physical activity outcomes 74 (Study 1, Fig. 4e).

Both *Choices* 64 and *Healthy Dads Healthy Kids* 69 cited the child as the agent of change (see Fig. 4e). For example, *Healthy Dads Healthy Kids* taught children to role model and encourage their fathers to adopt healthy behaviours, resulting in a significant change in both child and adult physical activity levels 69 (Study P, Fig. 4e). Further, authors of *Healthy Homework* report that intervention tasks were designed to foster family involvement, with the intended side effect of improving relationships and promoting healthier lifestyles throughout the family 45 (Study J, Fig. 4e).

The way in which the intervention was delivered was suggested to be important for engagement and efficacy (see Fig. 4f). Three different contexts for intervention delivery were identified. Evidence suggested that interventions tailored to the ethnic context within which they are delivered were well‐received (e.g. adherence to study protocol, engagement in intervention) 28, 31, 36, 86. The *Reach Out* intervention team took particular care to incorporate cultural characteristics from the African American community (the target group), adjusting the protocol to fit with language, food and activity preferences expressed by participants in formative qualitative research 36 (Study C, Fig. 4f). The importance of cultural relevance is further highlighted by *Aventuras Para Ninos* 28. The inclusion of Latino *promotoras* (health advisors who attended family homes to offer educational material and assistance with goal‐setting) was cited as critical to maintaining adherence to the intervention (which was targeted at Latino families) (Study B, Fig. 4f). Similarly, information provided by Chen and colleagues in an effective play‐based educational intervention was written in both Chinese and English, and delivered by bilingual counsellors 86 (Study T, Fig. 4f).

In addition to ensuring cultural relevance, Newton and colleagues suggest that targeting the whole family may be an effective strategy in increasing intervention adherence 77. Reflecting upon the pilot data from the *P‐Mobile* intervention, in which one parent and one child set goals for increasing their step counts, they state ‘that interventions could be strengthened by engaging both parents and incorporating siblings into the intervention’ (p11). Further research is needed to elucidate the impact of wider family engagement.

Rhodes and colleagues discuss the need to understand the specific barriers to physical activity experienced by target families before designing or evaluating an intervention 59. In a review of parenting that informed their later physical activity planning intervention, they report time constraints as most detrimental to health promotion 82. The synthesis identified two differing approaches to addressing parents' perceived lack of time (see Fig. 4f). The first focusses on facilitating social networks between families in the context of a participatory group‐based intervention 76. Parents were pleased that they were able to share project responsibilities, reducing the individual burden without compromising the efficacy of the intervention in increasing physical activity in their children (Study Y, Fig. 4f). In contrast, Chen and colleagues describe a mail‐based programme designed for time‐poor families, suggesting that the delivery of educational materials direct to family homes (therefore eradicating the need for families to schedule in intervention sessions) was effective in ensuring intervention adherence 86 (Study T, Fig. 4f).

## Discussion

This review showed that, overall, family‐based interventions to promote physical activity in children have been effective, with 66% demonstrating a positive effect on physical activity. Meta‐analysis on a subset of studies showed a small statistically significant effect in favour of the experimental group. The realist synthesis explored (where possible) aspects of the question ‘what works in family‐based physical activity promotion, for whom, under what circumstances, how, and why?’^26^. There was considerable heterogeneity in the strategies applied in the reviewed studies, but it was possible, nonetheless, to draw out patterns to inform future intervention efforts. Interventions employing goal‐setting and reinforcement techniques, and those focusing on the additional benefits of spending time physically active as a family, were highlighted as particularly useful. Clear articulation and evaluations of such strategies, taking into consideration the starting context of an intervention (particularly, family constraints, ethnicity and parental motivation), would add considerably to the evidence base.

### Intervention approaches supported by evidence

Our realist synthesis provided insight into strategies that may work to change children's physical activity behaviour within the context of families, highlighting the combination of goal‐setting and reinforcement as particularly effective. An increase in both parent and child motivation to change behaviour was identified as a potential *mechanism* (and an intermediate *outcome*), suggesting that identifying a target (e.g. a defined increase in steps per day 61), recording progress to provide feedback (e.g. using a log book 34), and rewarding achievement (e.g. receipt of equipment as prizes 49) may improve levels of motivation. This may, in turn, lead to a change in behaviour. This effect may be further amplified when a family spends time planning for physical activity (particularly useful in the context of family time constraints) 59. This finding is consistent with previous evidence of the positive effects of pedometer interventions 87 and of interventions that combine self‐monitoring with goal setting 88. It is also consistent with Self‐Regulation Theory 89, which posits that people regulate their behavior by comparing it with a standard (or goal), and taking action to change their behavior when they notice a significant disturbance, or discrepancy. Previous evidence suggests that 80% of parents incorrectly characterize their inactive child as ‘active’ 90, indicating a need for action. The work presented here extends this by showing that it is the combination of goal setting and reinforcement that appears to be most effective for families, particularly in the day‐to‐day context of families with limited time, work and school responsibilities and scheduling constraints.

A novel finding is that focusing an intervention on something *other than* physical activity for health or weight loss may also be a valuable approach. Effective studies in this review highlighted spending time with family, developing movement skills or improving self‐esteem rather than weight loss or other physical health indicators as desirable outcomes for intervention 42, 84. A focus on the *alternate* benefits of physical activity (as opposed to weight loss, for example) may encourage those typically stigmatized by obesity to volunteer for an intervention 91, 92. Enjoying time together, learning new skills and improving confidence, using physical activity as merely the vehicle for such change, may be more attractive to families who do not currently meet recommended physical activity guidelines 93. This is further supported by the observation that changes to the family psycho‐social environment may positively impact on physical activity behaviour (i.e. as the family becomes more engaged in active time together, family relationships may improve, resulting in greater enjoyment of time together, and subsequently, increased physical activity behaviour). As described above, ideas from the Family Systems Theory may support this virtuous cycle of behaviour 85, 94. This may be further enhanced by the child as the agent of change, where the child and their siblings drive the frequency of active time spent together as a family 64, 84. Based on the evidence from this review, it is not possible to assess the effect of family‐based interventions on physical activity levels of the other family members, or how much time is spent being active together. Exploring the potential displacement effect highlighted by the *C‐PET* intervention (in which the authors hypothesized that dog walks with the family may have replaced children's physical activity in other settings) may be an important avenue for future research 70.

### Intervention approaches not supported by evidence

In addition to highlighting effective strategies, this review identified approaches to behaviour change for which there is currently no evidentiary support. This is particularly important given the limited funding available for physical activity promotion, and the increasing need for evidence‐based policy 15. Despite evidence that education was effective in changing physical activity knowledge, education *alone* (e.g. studies providing mail‐based information about physical activity) was insufficient in changing behaviour 35, 41. Information‐based interventions that are unaccompanied by additional strategies may therefore be ineffective. Further appraisal of interventions targeting multiple health behaviours is also required, as we are currently unable to identify whether multi‐behaviour interventions are more or less effective in changing physical activity than those focused on physical activity only 16, 95.

### Insight into the importance of study context

Information from the systematic review and the realist synthesis offers further understanding of how best to intervene in specific ethnic groups. Tailoring the provision of physical activity interventions to the target population appeared particularly effective 28, 31, 36, 86. A previous review of physical activity interventions for children questioned the usefulness of such a strategy 16, calling specifically for further research to clarify whether targeting children from ethnic minority populations is beneficial (especially pertinent given consistently lower prevalence of physical activity in these groups 96, 97). Evidence from the current review indicates that in the context of family‐based interventions, adapting programme content to consider cultural preferences may be desirable. The use of culturally appropriate lay leaders to deliver interventions, for example, has shown to be efficacious 28. It is important to stress, however, that it remains unclear whether cultural tailoring is important in other intervention settings (such as schools).

Tailoring of interventions by other demographic characteristics was not a universal strategy. Single‐sex interventions were uncommon, with only seven studies specifically recruiting single‐sex child participants (all girls). These were more likely to be effective than mixed‐sex studies, supporting the conclusions of a recent meta‐analysis of interventions to promote physical activity in pre‐adolescent girls 98. However, without like‐for‐like comparisons of intervention strategies or consistent exploration of subgroup effects, it is difficult to draw robust conclusions on the effectiveness of this strategy. Previous evaluations of school‐based physical activity interventions have suggested that whilst both boys and girls may benefit, girls appear to benefit more 99. The difference in effectiveness between single sex and mixed‐sex studies in the current review may therefore be an artefact of the dilution of intervention effects when analysing both sexes combined. Future work should help elucidate this issue.

### Limitations of the evidence base

Based on the quality assessment, only three studies were of ‘strong’ methodological quality, and nearly half were rated ‘weak’. In particular, issues of selection bias were inadequately addressed. For example, 21% of studies did not report recruitment rates and therefore were unable to assess external validity. Subjective methods of measuring physical activity were employed in a relatively high number of studies, and long term follow‐up was uncommon. In addition, most studies were conducted in the USA; the generalizability of results from these studies to other countries is unclear. Further high quality research into family‐based physical activity promotion, with clear articulation of intended behaviour change mechanisms, is needed to strengthen the evidence.

### Review strengths and limitations

This review is the first to use the framework of a traditional systematic review to conduct a dual meta‐analysis *and* realist synthesis of family‐based interventions to promote physical activity in children 14. All study designs that reported on an evaluation of a family‐based physical activity intervention were included, allowing for a comprehensive appraisal of the evidence. Meta‐analyses allow for an objective appraisal of the evidence and provide an estimate of average treatment effect 100, 101, but are limited in their ability to describe *how* and *why* an intervention operates. In contrast, realist syntheses offer no quantifiable summary of intervention effectiveness, but consider the interaction between context, mechanism and outcome, exploring ‘what works for whom, under what circumstances, how, and why?’ 26. Previous reviews of physical activity promotion in children have been predominantly quantitative or narrative 15, 16, 18, 21, 24, and therefore these alone provide limited insight into the complex causal pathways that may underpin interventions. In addition to offering a more thorough assessment of published studies, this review supersedes existing reviews 15, 16, 18, 21, 24 by including substantially more interventions. A further strength of the review is the use of gold standard review methods (notably, duplication of screening, data extraction and study quality assessment).

This work is reliant on peer‐reviewed published data (rather than including grey literature). This may make the review vulnerable to publication bias 102, 103, and could have limited the representativeness of the programme theory. However, all relevant studies were included, irrespective of study design or quality, ensuring a comprehensive overview of the evidence base. In the realist synthesis, we only used data from the studies included in the systematic review to develop programme theory, although sibling papers (e.g. process evaluation papers) were sought, iterative searching of kinship papers was not conducted because of time constraints. This is likely to have limited our ability to report on complete CMO configurations, and fully elucidate *how and why* each intervention worked. However, given the novel dual approach used in this review, the results are more comprehensive than previous work, and will inform the development and dissemination of future family‐based interventions.

## Conclusions and recommendations

This combined review provides an up‐to‐date overview of the literature on physical activity promotion within family settings. Existing studies demonstrate a small effect on physical activity and, through a realist synthesis, highlight the following four key recommendations for practitioners and policy‐makers:

- Family‐based interventions should be tailored to the context within which they are delivered, most notably the ethnicity, motivation and time constraints of the family.
- Combining goal‐setting and reinforcement techniques should be considered to improve physical activity through increased motivation.
- Where a lack of resources and/or understanding for how to change behaviour exists, educational strategies should be employed to increase knowledge. However, these strategies should be combined with other intervention approaches to be successful in improving physical activity.
- Targeting improvements to the family psychosocial environment should be considered when designing interventions to increase both child and family physical activity. These should also include a focus on the child as the agent of change.

This review also identifies avenues for future research. To increase intervention engagement and efficacy, studies should examine the impact of greater emphasis on the psychological and social benefits of physical activity, of engaging wider family members, and of including a focus on developing children's movement skills. Moreover, studies should address issues of selection bias, study mediation effects to further elucidate causal pathways, include more detailed process evaluations, assess effects on other family members' physical activity and include long‐term follow‐up.

## Conflict of interest statement

There were no competing interests arising from this study.

## Supporting information


**Table S1:** Descriptive characteristics of studies included in systematic review of family‐based physical activity interventions.

**Table S2:** Results of duplicate quality assessment of studies, using the Effective Public Health Practice Project (EPHPP) Quality Assessment Tool for Quantitative Studies

**File S1:** Generic search terms, used in PubMed (title and abstract), Web of Knowledge (topic), Scopus (title, abstract and keywords), Ovid MEDLINE (abstract) and PsycInfo (abstract).

## References

[1] Ekelund U , Luan J , Sherar LB *et al.* Moderate to vigorous physical activity and sedentary time and cardiometabolic risk factors in children and adolescents. JAMA 2012; 307: 704–712. 22337681 10.1001/jama.2012.156 PMC3793121

[2] Hills AP , Andersen LB , Byrne NM . Physical activity and obesity in children. Br J Sport Med 2011; 45: 866–870. 10.1136/bjsports-2011-090199 21836171

[3] Lee I‐M , Shiroma EJ , Lobelo F *et al.* Effect of physical inactivity on major non‐communicable diseases worldwide: an analysis of burden of disease and life expectancy. Lancet 2012; 380: 219–229. 22818936 10.1016/S0140-6736(12)61031-9 PMC3645500

[4] Wilks DC , Sharp SJ , Ekelund U *et al.* Objectively measured physical activity and fat mass in children: a bias‐adjusted meta‐analysis of prospective studies. PLoS One 2011; 6: e17205. 21383837 10.1371/journal.pone.0017205 PMC3044163

[5] Summerbell C , Waters E , Edmunds L *et al.* Interventions for preventing obesity in children (Review) Interventions for preventing obesity in children. Heal San Fr 2009: 1–3.

[6] Boreham CA , McKay HA . Physical activity in childhood and bone health. Br J Sport Med 2011; 45: 877–879. 10.1136/bjsports-2011-090188 21807670

[7] Biddle SJH , Asare M . Physical activity and mental health in children and adolescents: a review of reviews. Br J Sport Med 2011; 45: 886–895. 10.1136/bjsports-2011-090185 21807669

[8] Brown HE , Pearson N , Braithwaite RE *et al.* Physical activity interventions and depression in children and adolescents : a systematic review and meta‐analysis. Sport Med 2013; 43: 195–206. 10.1007/s40279-012-0015-8 23329611

[9] Singh A , Uijtdewilligen L , Twisk JWR *et al.* Physical activity and performance at school: a systematic review of the literature including a methodological quality assessment. Arch Pediatr Adolesc Med 2012; 166: 49–55. 22213750 10.1001/archpediatrics.2011.716

[10] Janssen I , Leblanc AG . Systematic review of the health benefits of physical activity and fitness in school‐aged children and youth. Int J Behav Nutr Phys Act 2010; 7: 40. 20459784 10.1186/1479-5868-7-40 PMC2885312

[11] Andersen LB , Harro M , Sardinha LB *et al.* Physical activity and clustered cardiovascular risk in children: a cross‐sectional study (The European Youth Heart Study). Lancet 2006; 368: 299–304. 16860699 10.1016/S0140-6736(06)69075-2

[12] Nader PR , Bradley RH , Houts RM *et al.* Moderate‐to‐vigorous physical activity from ages 9 to 15 years. JAMA 2008; 300: 295–305. 18632544 10.1001/jama.300.3.295

[13] Dumith SC , Gigante DP , Domingues MR *et al.* Physical activity change during adolescence: a systematic review and a pooled analysis. Int J Epidemiol 2011; 40: 685–698. 21245072 10.1093/ije/dyq272

[14] Brown HE , Atkin AJ , Panter J *et al.* Family‐based interventions to increase physical activity in children: a meta‐analysis and realist synthesis protocol. BMJ Open 2014; 4: e005439. 10.1136/bmjopen-2014-005439 PMC4127934 25099934

[15] Metcalf B , Henley W , Wilkin T . Effectiveness of intervention on physical activity of children: systematic review and meta‐analysis of controlled trials with objectively measured outcomes (EarlyBird 54). BMJ 2012; 345: e5888. 23044984 10.1136/bmj.e5888

[16] Van Sluijs EMF , McMinn AM , Griffin SJ . Effectiveness of interventions to promote physical activity in children and adolescents: systematic review of controlled trials. BMJ 2007; 335: 703. 17884863 10.1136/bmj.39320.843947.BE PMC2001088

[17] Kipping RR , Howe LD , Jago R *et al.* Effect of intervention aimed at increasing physical activity, reducing sedentary behaviour, and increasing fruit and vegetable consumption in children: Active for Life Year 5 (AFLY5) school based cluster randomised controlled trial. BMJ 2014; 348: g3256. 24865166 10.1136/bmj.g3256 PMC4035503

[18] Van Sluijs EMF , McMinn A . Preventing obesity in primary schoolchildren. BMJ 2010; 340: c819. 20179127 10.1136/bmj.c819

[19] Gustafson SL , Rhodes RE . Parental correlates of physical activity in children and early adolescents. Sport Med 2006; 36: 79–97. 10.2165/00007256-200636010-00006 16445312

[20] McMinn AM , Griffin SJ , Jones AP *et al.* Family and home influences on children's after‐school and weekend physical activity. Eur J Public Heal Published Online First 2012; 23(5): 805–10 10.1093/eurpub/cks160 PMC3784797 23172732

[21] O'Connor TM , Jago R , Baranowski T . Engaging parents to increase youth physical activity a systematic review. Am J Prev Med 2009; 37: 141–149. 19589450 10.1016/j.amepre.2009.04.020

[22] Knowlden AP , Sharma M . Systematic review of family and home‐based interventions targeting paediatric overweight and obesity. Obes Rev 2012; 13: 499–508. 22221298 10.1111/j.1467-789X.2011.00976.x

[23] Barr‐Anderson DJ , Adams‐Wynn AW , DiSantis KI *et al.* Family‐focused physical activity, diet and obesity interventions in African–American girls: a systematic review. Obes Rev 2013; 14: 29–51. 23057473 10.1111/j.1467-789X.2012.01043.x PMC3524349

[24] Salmon J , Booth ML , Phongsavan P *et al.* Promoting physical activity participation among children and adolescents. Epidemiol Rev 2007; 29: 144–159. 17556765 10.1093/epirev/mxm010

[25] Van Sluijs EMF , Kriemler S , McMinn AM . The effect of community and family interventions on young people's physical activity levels: a review of reviews and updated systematic review. Br J Sport Med 2011; 45: 914–922. 10.1136/bjsports-2011-090187 PMC3736309 21836175

[26] Wong G , Greenhalgh T , Westhorp G *et al.* RAMESES publication standards: realist syntheses. BMC Med 2013; 11: 21. 23360677 10.1186/1741-7015-11-21 PMC3558331

[27] Cohen J . Statistical Power Analysis for the Behavioral Sciences (rev). Lawrence Erlbaum Associates, Inc: Hillsdale, NJ, USA, 1988.

[28] Crespo NC , Elder JP , Ayala GX *et al.* Results of a multi‐level intervention to prevent and control childhood obesity among Latino children: the Aventuras Para Ninos Study. Ann Behav Med 2012; 43: 84–100. 22215470 10.1007/s12160-011-9332-7 PMC4131843

[29] Pawson R . Evidence‐based policy: a realist perspective. Published Online First: 2006.

[30] Patterson TL , Sallis JF , Nader PR *et al.* Direct observation of physical activity and dietary behaviors in a structured environment: effects of a family‐based health promotion program. J Behav Med 1988; 11: 447–458. 3070048 10.1007/BF00844838

[31] Anand SS , Davis AD , Ahmed R *et al.* A family‐based intervention to promote healthy lifestyles in an aboriginal community in Canada. Can J Public Heal 2007; 98: 447–452. 10.1007/BF03405436 PMC6975761 19039880

[32] Bacardí‐Gascon M , Pérez‐Morales ME , Jiménez‐Cruz A *et al.* A six month randomized school intervention and an 18‐month follow‐up intervention to prevent childhood obesity in Mexican elementary schools. Nutr Hosp 2012; 27: 755–762. 23114940 10.3305/nh.2012.27.3.5756

[33] Baker J , Saunders K . Fitter, healthier, happier families: a partnership to treat childhood obesity in the West Midlands. J Public Heal 2012; 126: 332–334. 10.1016/j.puhe.2012.01.013 22410392

[34] Barr‐Anderson D , Adams‐Wynn A , Alhassan S *et al.* Culturally‐appropriate, family‐ and community‐based physical activity and healthy eating intervention for African–American middle school‐aged girls: a feasibility pilot. J Adolesc Fam Heal 2014; 6: 2(6).

[35] Beech B , Klesges R . Child‐and parent‐targeted interventions: the Memphis GEMS pilot study. Ethn Dis Published Online First 2003 Winter;13(1 Suppl. 1): S40–53 12713210

[36] Burnet DL , Plaut AJ , Wolf SA *et al.* Reach‐out: a family‐based diabetes prevention program for African American youth. J Natl Med Assoc 2011; 103: 269–277. 21671531 10.1016/s0027-9684(15)30290-x

[37] Catenacci V , Barrett C . Changes in physical activity and sedentary behavior in a randomized trial of an internet‐based versus workbook‐based family intervention study. J Phys Act Heal Published Online First 2014; 11(2): 348–358. 10.1123/jpah.2012-0043 PMC4570826 23364318

[38] Centis E , Marzocchi R , Di Luzio R *et al.* A controlled, class‐based multicomponent intervention to promote healthy lifestyle and to reduce the burden of childhood obesity. Pediatr Obes 2012; 7: 436–445. 22911919 10.1111/j.2047-6310.2012.00079.x

[39] Chen J‐LL , Weiss S , Heyman MB *et al.* The efficacy of the web‐based childhood obesity prevention program in Chinese American adolescents (Web ABC study). J Adolesc Heal 2011; 49: 148–154. 10.1016/j.jadohealth.2010.11.243 PMC3143380 21783046

[40] Chen JL , Weiss S , Heyman MB *et al.* Efficacy of a child‐centred and family‐based program in promoting healthy weight and healthy behaviors in Chinese American children: a randomized controlled study. J Public Heal 2010; 32: 219–229. 10.1093/pubmed/fdp105 PMC2875985 19933120

[41] Chen JL , Weiss S , Heyman MB *et al.* Pilot study of an individually tailored educational program by mail to promote healthy weight in Chinese American children. J Spec Pediatr Nurs 2008; 13: 212–222. 18638051 10.1111/j.1744-6155.2008.00155.x PMC2877702

[42] Cliff DP , Okely AD , Morgan PJ *et al.* Movement skills and physical activity in obese children: randomized controlled trial. Med Sci Sport Exerc 2011; 43: 90–100. 10.1249/MSS.0b013e3181e741e8 20473216

[43] Coppins DF , Margetts BM , Fa JL *et al.* Effectiveness of a multi‐disciplinary family‐based programme for treating childhood obesity (the Family Project). Eur J Clin Nutr 2011; 65: 903–909. 21487425 10.1038/ejcn.2011.43

[44] Delamater AM , Pulgaron ER , Rarback S *et al.* Web‐based family intervention for overweight children: a pilot study. Child Obes 2013; 9: 57–63. 23308372 10.1089/chi.2011.0126 PMC3621342

[45] Duncan S , McPhee JC , Schluter PJ *et al.* Efficacy of a compulsory homework programme for increasing physical activity and healthy eating in children: the healthy homework pilot study. Int J Behav Nutr Phys Act 2011; 8: 127. 22085440 10.1186/1479-5868-8-127 PMC3256102

[46] Estabrooks PA , Shoup JA , Gattshall M *et al.* Automated telephone counseling for parents of overweight children: a randomized controlled trial. Am J Prev Med 2009; 36: 35–42. 19095163 10.1016/j.amepre.2008.09.024

[47] Finkelstein EA , Tan YT , Malhotra R *et al.* A Cluster Randomized Controlled Trial of an Incentive‐Based Outdoor Physical Activity Program. J Pediatr Published Online First, 2013; 163(1): 167–72.e1. 10.1016/j.jpeds.2013.01.009 23415616

[48] Golley RK , Magarey AM , Daniels LA . Children's food and activity patterns following a six‐month child weight management program. Int J Pediatr Obes 2011; 6: 409–414. 21838569 10.3109/17477166.2011.605894

[49] Greening L , Harrell KT , Low AK *et al.* Efficacy of a school‐based childhood obesity intervention program in a rural southern community: TEAM Mississippi Project. Obes (Silver Spring) 2011; 19: 1213–1219. 10.1038/oby.2010.329 21233806

[50] Hardman CAC , Horne PPJ , Lowe CF . A home‐based intervention to increase physical activity in girls: The ‘fit n fun’ dudes program. J Exerc Sci Fit 2009; 7: 1–8.

[51] Hovell MF , Nichols JF , Irvin VL *et al.* Parent/Child training to increase preteens' calcium, physical activity, and bone density: a controlled trial. Am J Heal Promot 2009; 24: 118–128. 10.4278/ajhp.08021111 PMC3571764 19928484

[52] Jacobson D , Melnyk BM . A primary care healthy choices intervention program for overweight and obese school‐age children and their parents. J Pediatr Heal Care 2012; 26: 126–138. 10.1016/j.pedhc.2010.07.004 22360932

[53] Jago R , Sebire SJ , Turner KM *et al.* Feasibility trial evaluation of a physical activity and screen‐viewing course for parents of 6 to 8 year‐old children: Teamplay. Int J Behav Nutr Phys Act 2013; 10: 31. 23510646 10.1186/1479-5868-10-31 PMC3598924

[54] Nader PPR , Sallis JJF , Abramson IS *et al.* Family‐based cardiovascular risk reduction education among Mexican and Anglo‐Americans. Fam Community … 1992; 15: 57–58.

[55] Nader PR , Sallis JF , Patterson TL *et al.* A family approach to cardiovascular risk reduction: results from the San Diego Family Health Project. Health Educ Q 1989; 16: 229–244. 2732065 10.1177/109019818901600207

[56] Olvera N , Bush JA , Sharma SV *et al.* BOUNCE: a community‐based mother‐daughter healthy lifestyle intervention for low‐income Latino families. Obes (Silver Spring) 2010; 18(Suppl. 1): S102–S104. 10.1038/oby.2009.439 20107454

[57] Olvera N , Scherer R , McLeod J *et al.* BOUNCE: an exploratory healthy lifestyle summer intervention for girls. Am J Heal Behav 2010; 34: 144–155. 10.5993/ajhb.34.2.2 19814594

[58] Ransdell LB , Robertson L , Ornes L *et al.* Generations Exercising Together to Improve Fitness (GET FIT): a pilot study designed to increase physical activity and improve health‐related fitness in three generations of women. Women Heal 2004; 40: 77–94. 10.1300/j013v40n03_06 15829447

[59] Rhodes RE , Naylor P‐JJ , McKay HA . Pilot study of a family physical activity planning intervention among parents and their children. J Behav Med 2010; 33: 91–100. 19937106 10.1007/s10865-009-9237-0

[60] Rodearmel SJ , Wyatt HR , Barry MJ *et al.* A family‐based approach to preventing excessive weight gain. Obesity (Silver Spring) 2006; 14: 1392–1401. 16988082 10.1038/oby.2006.158

[61] Rooney BL , Gritt LR , Havens SJ *et al.* Growing healthy families: family use of pedometers to increase physical activity and slow the rate of obesity. WMJ 2005; 104: 54–60. 16138517

[62] Schwartz RP , Vitolins MZ , Case LD *et al.* The YMCA Healthy, Fit, and Strong Program: a community‐based, family‐centered, low‐cost obesity prevention/treatment pilot study. Child Obes 2012; 8: 577–582. 23181924 10.1089/chi.2012.0060 PMC6463988

[63] Shelton D , Le Gros K , Norton L *et al.* Randomised controlled trial: a parent‐based group education programme for overweight children. J Paediatr Child Heal 2007; 43: 799–805. 10.1111/j.1440-1754.2007.01150.x 17854421

[64] Siwik V , Kutob R , Ritenbaugh C *et al.* Intervention in overweight children improves body mass index (BMI) and physical activity. J Am Board Fam Med 2013; 26: 126–137. 23471926 10.3122/jabfm.2013.02.120118 PMC4010584

[65] Story M , Sherwood NE , Himes JH *et al.* An after‐school obesity prevention program for African–American girls: the Minnesota GEMS pilot study. Ethn Dis 2003; 13: S54–S64. 12713211

[66] Towey M , Harrell R , Lee B . Evaluation of ‘one body, one life’: a community‐based family intervention for the prevention of obesity in children. J Obes 2011; 2011: 619643. 22028958 10.1155/2011/619643 PMC3199086

[67] Trost SG , Tang R , Loprinzi PD . Feasibility and efficacy of a church‐based intervention to promote physical activity in children. J Phys Act Heal 2009; 6: 741–749. 10.1123/jpah.6.6.741 20101917

[68] Nyberg G , Sundblom E , Norman Å *et al.* Effectiveness of a universal parental support programme to promote healthy dietary habits and physical activity and to prevent overweight and obesity in 6‐year‐old children: the healthy school start study, a cluster‐randomised controlled trial. PLoS One n/d; 10: e0116876 2015. 25680096 10.1371/journal.pone.0116876 PMC4332680

[69] Morgan PJ , Collins CE , Plotnikoff RC *et al.* The ‘Healthy Dads, Healthy Kids’ community randomized controlled trial: a community‐based healthy lifestyle program for fathers and their children. Prev Med (Baltim) 2014; 61: 90–99. 10.1016/j.ypmed.2013.12.019 24380796

[70] Morrison R , Reilly JJ , Penpraze V *et al.* Children, parents and pets exercising together (CPET): exploratory randomised controlled trial. BMC Public Health 2013; 13: 1096. 24279294 10.1186/1471-2458-13-1096 PMC4222564

[71] Smith LR , Chadwick P , Radley D *et al.* Assessing the short‐term outcomes of a community‐based intervention for overweight and obese children: The MEND 5‐7 programme. BMJ Open 2013; 3 (5): pii: e002607. 10.1136/bmjopen-2013-002607 PMC3646180 23645925

[72] Newton RL , Marker AM , Tudor‐Locke C *et al.* Promoting physical activity in children using a parent‐targeted mobile phone intervention. Ann Behav Med 2014; 47: S171. 10.2196/mhealth.3420 PMC4260004 25386899

[73] Arredondo EM , Morello M , Holub C *et al.* Feasibility and preliminary findings of a church‐based mother–daughter pilot study promoting physical activity among young Latinas. Fam Community Health 2014; 37: 6–18. 24297004 10.1097/FCH.0000000000000015

[74] Eather N , Morgan PJ , Lubans DR . Feasibility and preliminary efficacy of the Fit4Fun intervention for improving physical fitness in a sample of primary school children: a pilot study. Phys Educ Sport Pedagog 2013; 18: 389–411.

[75] Higgins JPT , Thompson SG , Deeks JJ *et al.* Measuring inconsistency in meta‐analyses. BMJ 2003; 327: 557–560. 12958120 10.1136/bmj.327.7414.557 PMC192859

[76] De Bock F , Genser B , Raat H *et al.* A participatory physical activity intervention in preschools: a cluster randomized controlled trial. Am J Prev Med 2013; 45: 64–74. 23790990 10.1016/j.amepre.2013.01.032

[77] Newton RLJ , Marker AM , Allen HR *et al.* Parent‐targeted mobile phone intervention to increase physical activity in sedentary children: randomized pilot trial. J Med INTERNET Res 2014; 16: e48. 25386899 10.2196/mhealth.3420 PMC4260004

[78] Ajzen I . From intentions to actions In: Attitudes, Personality, & Behavior, 1988, pp. 1–21.

[79] Madden TJ , Ellen PS , Ajzen I . A comparison of the theory of planned behavior and the theory of reasoned action. Personal Soc Psychol Bull 1992; 18: 3–9.

[80] Fishbein M . A theory of reasoned action: some applications and implications. Nebraska Symp Motiv 1979; 27: 65–116. 7242751

[81] Schwarzer R , Lippke S , Ziegelmann JP . Health action process approach. Zeitschrift für Gesundheitspsychologie 2008; 16: 157–160.

[82] Bellows‐Riecken KH , Rhodes RE . A birth of inactivity? A review of physical activity and parenthood. Prev Med 2008; 46: 99–110. 17919713 10.1016/j.ypmed.2007.08.003

[83] Bandura A . Social cognitive theory of self‐regulation. Organ Behav Hum Decis Process 1991; 50: 248–287.

[84] Morgan PJ , Lubans DR , Callister R *et al.* The ‘Healthy Dads, Healthy Kids’ randomized controlled trial: efficacy of a healthy lifestyle program for overweight fathers and their children. Int J Obes (Lond) 2011; 35: 436–447. 20697417 10.1038/ijo.2010.151

[85] Kerr ME . Family systems theory and therapy In: Handbook of Family Therapy, 1981, pp. 226–264.

[86] Chen J‐L , Weiss S , Heyman MB *et al.* Efficacy of a child‐centred and family‐based program in promoting healthy weight and healthy behaviors in Chinese American children: a randomized controlled study. J Public Health (Oxf) 2010; 32: 219–229. 19933120 10.1093/pubmed/fdp105 PMC2875985

[87] Bravata DM , Smith‐Spangler C , Sundaram V *et al.* Using pedometers to increase physical activity and improve health: a systematic review. JAMA 2007; 298: 2296–2304. 18029834 10.1001/jama.298.19.2296

[88] Michie S , Hardeman W , Fanshawe T *et al.* Investigating theoretical explanations for behaviour change: the case study of ProActive. Psychol Heal 2007; 23(1): 25–39. 10.1080/08870440701670588 25159905

[89] Carver CS , Scheier MF . On the Self‐Regulation of Behavior. Cambridge University Press: Cambridge, UK, 2001.

[90] Corder K , van Sluijs E , McMinn AM *et al.* Perception versus reality awareness of physical activity levels of British children. Am J Prev Med 2010; 38: 1–8. 20117551 10.1016/j.amepre.2009.08.025 PMC3746297

[91] Puhl R , Brownell KD . Bias, discrimination, and obesity. Obes Res 2001; 9: 788–805. 11743063 10.1038/oby.2001.108

[92] Puhl RM , Heuer CA . The stigma of obesity: a review and update. Obesity 2009; 17: 941–964. 19165161 10.1038/oby.2008.636

[93] Department of Health Improvement and Protection PA . Start active, stay active: a report on physical activity from the four home countries' chief medical officers. 2011.

[94] Krumeich A , Weijts W , Reddy P *et al.* The benefits of anthropological approaches for health promotion research and practice. Heal Educ Res 2001; 16: 121–130. 10.1093/her/16.2.121 11345657

[95] Prochaska JOJJ , Nigg CR , Spring B *et al.* The benefits and challenges of multiple health behavior change in research and in practice. Prev Med (Baltim) 2010; 50: 26–29. 10.1016/j.ypmed.2009.11.009 PMC2813890 19948184

[96] Gordon‐Larsen P , McMurray RG , Popkin BM . Adolescent physical activity and inactivity vary by ethnicity: The National Longitudinal Study of Adolescent Health. J Pediatr 1999; 135: 301–306. 10484793 10.1016/s0022-3476(99)70124-1

[97] Sallis JF , Zakarian JM , Hovell MF *et al.* Ethnic, socioeconomic, and sex differences in physical activity among adolescents. J Clin Epidemiol 1996; 49: 125–134. 8606313 10.1016/0895-4356(95)00514-5

[98] Biddle SJH , Braithwaite R , Pearson N . The effectiveness of interventions to increase physical activity among young girls: a meta‐analysis. Prev Med 2014; 62: 119–131. 24530611 10.1016/j.ypmed.2014.02.009

[99] Grydeland M , Bergh IH , Bjelland M *et al.* Intervention effects on physical activity: the HEIA study—a cluster randomized controlled trial. Int J Behav Nutr Phys Act 2013; 10: 17. 23379535 10.1186/1479-5868-10-17 PMC3598379

[100] Egger M , Smith GD . Meta‐analysis. Potentials and promise. BMJ 1997; 315: 1371–1374. 9432250 10.1136/bmj.315.7119.1371 PMC2127866

[101] Riley RD , Higgins JPT , Deeks JJ . Interpretation of random effects meta‐analyses. BMJ 2011; 342: d549. 21310794 10.1136/bmj.d549

[102] Dickersin K . The existence of publication bias and risk factors for its occurrence. JAMA 1990; 263: 1385–1389. 2406472

[103] Easterbrook PJ , Berlin JA , Gopalan R *et al.* Publication bias in clinical research. Lancet 1991; 337: 867–876. 1672966 10.1016/0140-6736(91)90201-y

