# Supplementary material for: Family‐based interventions to increase physical activity in children: a systematic review, meta‐analysis and realist synthesis
Source: Obes Rev. 2016 Jan 12;17(4):345–60. doi: 10.1111/obr.12362 (PMC4819691; doi:10.1111/obr.12362)
Supplement: Supplementary file 1 — Table S1: Descriptive characteristics of studies included in systematic review of family‐based physical activity interventions. [file OBR-17-345-s001.docx]

*Supplementary* Table 1: Descriptive characteristics of studies included in systematic review of family-based physical activity interventions.

| **Intervention name**  **Study**  (first author; year of publication; country)  **Mode of synthesis** | **Design**  (study design; level of randomisation where applicable) | **Participants**  (n children analysed, and percentage of those first contacted; mean years of age ± SD at baseline; % male; mean weight status at baseline) | **PA measurement** (assessment points; measure used; outcomes reported) | **Intervention**  (intervention duration; delivery; theoretical grounding) | **Intervention**  (intervention dose; content; control) | **Outcome**  (summary statement)  **Data reported**  (PA change; statistical tests reported) |
| --- | --- | --- | --- | --- | --- | --- |
| **The Healthy School Start Study**(1)  Nyberg; 2015; USA  Narrative review, meta-analysis, realist synthesis | CRCT; pre-school class | N=241; 39%  Age: 6.2 ± 0.3  % male: 51  Weight status: 77.2% healthy weight (19.1% overweight/ obese, 3.7% underweight) | Baseline, 6m, 12m; objective (ActiGraph GT3X+ accelerometer); total PA (cpm) and total/ weekend minutes of MVPA | 6m; motivational interviews were delivered with trained counsellors and health education/classroom activities by school teachers; theory: Social Cognitive Theory (plus programme theory) | Parents in the intervention group were offered two 45min motivational interviewing sessions, which targeted parental care and control, self-efficacy to provide support for healthy eating and PA. Children and their parents were provided with 10 30min classroom activities (with accompanying homework) aimed at their knowledge, attitudes, and preferences. Families also received a detailed health information brochure on encouraging healthy PA and dietary practices. Control classes were offered the whole programme directly after the 6-months follow-up measurements. | **Did not favour intervention**  At T2 (6m), there were no significant differences in PA between groups (total cpm or MVPA, either for the whole week or at the weekend). |
| **No name reported**(2)  Arredondo; 2014; USA  Narrative review, realist synthesis | *Pilot* trial (one group, pre- and post- measures); N/A | N=10; 91%  Age: 9.6 ± 1.1  % male: 0  Weight status: not reported | Baseline, 2m; parent-reported (questionnaire not reported); frequency of PA per week | 8wk; delivered by promotora (Spanish-speaking) and principal investigator; theory: Social Cognitive Theory and Family Systems Theory | Participating mothers and daughters attended weekly sessions (2.5 hours). Sessions consisted of PA and nutrition education, reinforced by interactive activities and PA, and incorporating religious themes. No control group. | **Favoured intervention**  Reported mean PA hours per week increased from 6.25 ± 3.66 pre-intervention, to 7.68 ± 3.76 post-intervention. No p-values reported. |
| **P-Mobile**(3)  Newton; 2014; USA  Narrative review, realist synthesis | RCompT; children | N=27; 46%  Age: 8.7 ± 1.4  % male: 44  Weight status: BMI percentile 85.6 | Baseline, 3m; objective (NL-1000 pedometer); steps per day | 12wk; delivered via a mobile phone application and website; theory: Social Cognitive Theory | Parents in the Minimal Intervention Group (MIG) were given access to a website (formatted by mobile phone use) in which they could view their child’s daily step count, monitor their child’s step counts, and receive monthly nutrition tips. Parents in the Intensive Intervention Group (IIG) received an extended version of the website, in addition to access to articles focused on behavioural strategies to increase PA, and regular text message prompts. | **Favoured intervention**  Children in both the MIG and the IIG significantly increased their PA over 12 weeks, by 1427.6 ± 583.0 (p=0.02) and 2832.8 ± 604.9 (p=<0.001) respectively. There were no statistically significant differences between the groups. |
| **Healthy Dads Healthy Kids**(25)  Morgan; 2014: Australia  Narrative review, meta-analysis, realist synthesis | RCT; families | N=132; 83%  Age: 8.1 ± 2.1  % male: 55.0  Weight status: 66% healthy weight (23% overweight, 10% obese and 1% underweight) | Baseline, 3m, 6m; objective (Yamax SW-700 pedometer); steps/day | 3m; delivered by authors (described as qualified teachers with expertise in physical education); theory: Social Cognitive Theory and Family Systems Theory | Fathers attended 8 face-to-face 75min sessions - of which five were education for fathers only - focussing on PA and dietary behaviour. Emphasized modelling, reinforcing and providing opportunities/removing barriers to PA, plus information of facilitating healthy diet choices. Children attended three active sessions, containing fundamental movement skills, rough and tumble play, health-related fitness and 'fun/active' games. PA handbook, weight loss handbook (for fathers). A program folder with session objectives, and access to free public website to self-monitor during program, were provided. Cross-over control design ensured participants received the intervention 6m after baseline. | **Favoured intervention**  Significant group x time differences for mean steps per day; those in intervention group increased by 1468 (95% CI 631, 2305) compared to reduction of 157 (95% CI-10.28, 713); p=0.01. |
| **No name reported** (4,5)  De Bock; 2013; Germany  Narrative review, meta-analysis, realist synthesis | CRCT; pre-school class | N=803; 78%  Age: 5.1 (SD not reported)  % male: 52  Weight status: 5.4% overweight | Baseline, 6m, 12m; objective (Actiheart accelerometer); mean counts per minute | 6m; ideas delivered via a project website; theory: General Systems Theory | Parents were given access to a study website with ideas for encouraging PA, focusing on active transport (walking bus), ‘lifestyle’ PA (gardening club), promotion of outdoor activities (forest trips), and reducing obesogenic traditions (e.g. healthier birthday parties). Parents were able to add suggestions to the website, and tailor to their own setting, ensuring a participatory approach was adopted. The control arm received the non-participatory state-sponsored PA program. | **Favoured intervention**  Children in the intervention group reported significantly higher mean PA levels (increase of 1.38 counts per 15 second interval; 95% CI 0.22, 2.54, p=0.019) at 6m. Time in MVPA was not different at 12m. |
| **Fit4Fun**(6)  Eather; 2013; Australia  Narrative review, meta-analysis, realist synthesis | RCT; schools | N=46; 82%  Age: 10.9 ± 0.67  % male: 40  Weight status: not reported | Baseline, 10wk; objective (Yamax SW700 pedometer); steps per day | 8wk; delivered by research team; theory: Social Cognitive Theory and Harter’s Competence Motivation Theory | Children in the intervention group received an 8wk curriculum of health/PE classes, designed to improve knowledge, skills, and health-related fitness (HRF) understanding. Families were also provided with an ‘enjoyable and engaging’ home PA programme (fitness activities, small-sided games, and challenges), which aimed to improve their HRF levels. Goal-setting activities were included to monitor progress. Schools were provided with activity task cards to allow for optional vigorous games during break time, and incentives for completion of the home fitness programme. The control group participated in their usual 60 min/week HPE lesson over the 8-week intervention period. | **Did not favour intervention**  No statistically significant group x time effects were found for PA (mean steps per day). |
| **CPET: Children and Pets Exercising Together**(7)  Morrison; 2013; UK  Narrative review, meta-analysis, realist synthesis | RCT; families | N=30; 21%  Age: 10.9 (SD not reported)  % male: 33  Weight status: 80% healthy weight, 13% overweight, 7% obese | Baseline, 11wk; objective (ActiGraph GT3X+ accelerometer); mean accelerometer counts per minute, % time spent in PA | 10wk; delivered by animal behaviourist and PA research assistant; theory: none | Intervention families received 3 home visits (1 from an animal behaviourist and 2 from a PA research assistant), in addition to regular phone calls and text messages to review goal progress, address questions, and provide encouragement. Information on dog walking routes and active play with the dog was provided to encourage parents, children and the family dog to be physically active together. The control group received no intervention. | **Did not favour intervention**  There were no significant difference in the total volume of PA, amount of time in sedentary or light intensity PA, or MVPA (p-values ranged from 0.12 to 0.41). |
| **MEND 5-7** (8)  Smith; 2013; UK  Narrative review, realist synthesis | Trial (one group, pre- and post-measures); N/A | N=440; not reported  Age: 6.1 ± 0.8  % male: 42  Weight status: mean BMI 22.5 ± 3.6, BMI z-score 2.86 ± 0.91 | Baseline, 10wk; parent-reported (questionnaire adapted from the ‘outdoor playtime checklist’); hrs participating in PA/week | 10wk; delivered by local staff from a variety of backgrounds (including healthcare, PA and other professionals); theory: none | 10 1hr 45min group sessions, held in community settings, including behaviour change, nutrition and PA education. No control group. | **Favoured intervention**  Mean hours participating in PA/week increased significantly from pre-to –post intervention (+2.9hr/week p=<0.01). |
| **AOM: America on the Move**(9)  Catenacci; 2013; USA  Narrative review only | RCompT; families | N=131; 71%  Age:10.5 ± 1.4  % male: 51  Weight status: BMI 18.0 ± 3.3 | Baseline, 6wk, 3m; objective (ActiCal accel); time spent in PA and sedentary behaviour | 12wk; delivered remotely (either workbook or internet); theory: none | All participants given pedometers and advised to increase steps relative to an established baseline. Weekly emails reminded both groups (internet and workbook) to record steps and continue progressing with goals. No control group. | **Did not favour intervention**  When adjusted for age and sex, there were no differences in light, moderate, vigorous or MVPA between groups at baseline, wk 6 or wk 12 (p-values ranged from 0.52 to 0.96). |
| **Choices**(10)  Siwik; 2013: USA  Narrative review only | Wait-list RCT; children | N=22; 29%  Age: 9.6 ± 0.6  % male: 47  Weight status: BMI 26.6 ± 5.2 | Baseline, 3m, 6m, 9m, 12m, 15m; self-reported (48-hour PA recall); time spent in high MET activities | 3m; delivered by family physician (parents) or family medicine residents (children); theory: none | Participants met weekly for 90min session, organised into 20-30min segments (individual and group check-in, class content and PA). Class content included benefits, barriers and facilitators of PA, resilience/coping, fast foods, television viewing. Participants encouraged to modify their lifestyle individually in relationship to their goals. Lagged intervention ensured all participants received intervention. | **Favoured intervention**  Large estimated intervention effect for increasing high-MET activities (1.82 METs, SE 1.42), and similarly large reduction effect for low-MET activities (-1.04 METs, SE 0.60) (medium-MET activities remained unchanged; 0.13 METs SE 1.08). Net shift of activity from low-MET to high-MET had significant intervention effect of 2.84 METs (SE 1.36). |
| **A Family Affair**(11)  Barr-Anderson; 2013 (in press); USA  Narrative review, realist synthesis | *Pilot* trial (one group, pre- and post- measures); N/A | N=8; 67%  Age: 12.4 ± 1.4  % male: girls only  Weight status: mean BMI percentile 92.6 ± 12.8 | Baseline, 3m, 9m; self-reported (questionnaire not reported); MVPA min/day | Phase One 8wk and Phase Two 6m; delivered by female African-American instructors; theory: none | Phase One included 8 3hr weekly face-to-face sessions with instructor-led physical activity, post-workout healthy snack, interactive nutrition education class and goal-setting/problem-solving sessions with pedometers and rewards for achievement of goals, Phase Two (maintenance) included 6m of monthly newsletters reinforcing behavioural change information (with extra monthly session in the final two months). Mother-daughter pairs also participated in three family events/celebrations. No control group. | **Favoured intervention**  MVPA min/day increased from baseline, to end of Phase One, and end of Phase Two (22.7 SD 26.56, to 30.2 SD 28.32, to 40.9 SD 27.36). Active video games hours/day increased from baseline to end of Phase One (1.7 SD 1.91 to 2.2 SD 1.15), decreased again at the end of Phase Two (1.8 SD 2.19); p-values not reported. |
| **Fit for Health**(12)  Delamater; 2013: USA  Narrative review, realist synthesis | *Pilot* trial (two groups, pre- and post-measures); N/A | N=18; 75%  Age: 11.0 ± 1.2  % male: 58  Weight status: mean BMI percentile 98% | Baseline, 1m; self-reported (BRFSSS); frequency of PA and SB | 1m; delivered online; theory: none | Intervention featured online games discriminating PA from SB, and healthy from unhealthy foods/portion sizes. Other modules addressed stimulus control and modification of eating behaviour, increasing PA and reducing SB. Children also identified 3 goals (1: step count, tracked using provided pedometer, 2: a self-selected PA/SB and 3: a self-selected dietary behaviour). Goals were tracked using website. Parents given additional educational materials via website, and encouraged to monitor, prompt and reinforce use of the program. No control group. | **Favoured intervention**  Significant effects (over time, regardless of website usage) were observed for PA – change not reported, no table provided: in text figures (F(1, 16) = 7.58, p=0.005). |
| **Teamplay**(13)  Jago; 2013; UK  Narrative review, meta-analysis | *Pilot* RCT; parents | N=38; 51%  Age: 7.3 ± 1.6  % male: 38.1 (intervention) 31.3 (control)  Weight status: not reported | Baseline, 8wk, 12wk; objective (ActiGraph GT1M accelerometer); MVPA, accelerometer counts/min | 2m; delivered by two members of research team who had received Parent Group Leader training from Family Links; theory: Social Determination Theory | Parents attended 8 weekly sessions (2hr each) at local community centres, without their children. Each session made up of 3 main topics, plus time for refreshments, games, parent feedback and introduction of tasks to be completed at home. Content covered strategies for increasing PA and reducing SV, plus facilitation information (praise, expectations, consistency, problem solving). The control group received no additional input during the period of the intervention, but was provided with written materials summarizing the intervention content at the end of the study. | **Mixed evidence**  Intervention group engaged in 2.6 fewer minutes of weekday MVPA (8wk), but 11 more minutes at the weekend (8wk) than the control group (similar pattern observed for accel counts; statistical significance not tested as pilot study). |
| **No name reported**(14)  Finkelstein; 2013; Singapore  Narrative review, meta-analysis, realist synthesis | RCT; families | N=234; 47%  Age: 8.2 ± 1.5  % male: 54  Weight status: BMI 24 ± 3.4 | Baseline, 9m (range: 6-10m); objective (Omron pedometer); steps/wk, and by week and weekend day | 6-10m; delivery not reported; theory: none | Families received educational materials (benefits of PA), and information about available weekend outdoor activities and pedometer step targets. Pedometers were provided to record daily steps; those children meeting 8000 steps/day were rewarded with vouchers. Those meeting step targets and attending outdoor activities were also eligible to win larger prizes (e.g. zoo tickets). The control group received health promotion information, and were offered intervention post-completion of study period. | **Favoured intervention**  Children in intervention group logged more steps/wk (8660 vs. 7767 for control group), during weekdays (8646 vs. 7826) and on weekends (8779 vs. 7684). Over 7-day period, 24% intervention group achieved 8000 steps/day, compared with only 1.9% controls. Difference more pronounced at weekends (50.0% vs. 12.6%) than on weekdays (33.0% vs. 8.4%). Difference in % attaining 8000 steps/day over week: 15.72 (p=0.001), on weekdays 5.87 (0.006) and at weekends 29.32 (0.003). |
| **The Aventuras Para Ninos Study**(15)  Crespo; 2012; USA  Narrative review, meta-analysis, realist synthesis | CRCT; schools | N=796; 55%  Age: 5.9 ± 0.9 % male: 50 Weight status: 17% overweight and 30% obese | Baseline, 12m, 24m, 36m; parent-reported (questionnaire not reported); PA and sports over past yr | 12m; delivered by 'Promotoras' (trained female diet and PA counsellors); theory: Health Belief Model and Social Cognitive Theory (family), Structural Model of Health Behaviour (community) | Three intervention conditions were included: family, community and a ‘combined’ condition. Family intervention involved home visits (newsletters around PA, healthy eating recipes, discussion of PA barriers, strategies and goal-setting) plus regular 'booster' phone calls. Community intervention included improvements to school playgrounds and community parks, changes to classroom practices and school meals, plus additional PE equipment. The control group received no intervention. | **Favoured intervention**  Family-only intervention increased parent-reported child PA (baseline: 2.98, 12m: 3.28, 24m: 3.36, 36m: 3.15) - score of 1 to 5. Family main effect for PA beta 0.21 (SE 0.078) p=0.008 (community p=0.24). |
| **Healthy Choices Intervention**(16)  Jacobson; 2012: USA  Narrative review, realist synthesis | Trial (one group, pre- and post-measures); N/A | N=71; 95%  Age:10.8 ± 1.3  % male: 35  Weight status: BMI 26.1 ± 3.1 | Baseline, 7wk; self-reported (HLBS); Likert scale of involvement in healthy lifestyle behaviours | 7-13wk; delivery not reported; theory: Cognitive Theory | Intervention design adapted from the COPE/Healthy Lifestyles TEEN (Thinking, Emotions, Exercise, and Nutrition) Intervention Program, and focused on cognitive behaviour skills building, increasing knowledge and beliefs, parental involvement, and psychosocial content - children and parents received Healthy Choices program weekly contacts (four personalised face-to-face 30min-1 hour clinic sessions alternated with three 30-45 min telephone sessions). No control group. | **Favoured intervention**  Mean difference in 60min/day activity (after intervention) -1.13 (information not provided as to units or clinical significance), p=0.52, 95CI% -2.07, -0.19. |
| **Healthy, Fit and Strong**(17)  Schwartz; 2012; USA  Narrative review only | Feasibility study; N/A | N=53: 90%  Age: 42% 6-8yr, 58% 9-11yr (mean or SD not reported)  % male: 37 Weight status:14% 85-94^th^, 42% 95-98^th^, 44% above 99^th^ BMI percentile (median BMI z-score 2.19, range 1.06-3.51; mean and SD not reported) | Baseline, 3m, 6m, 12m; brief interviewer-delivered questionnaire (developed by the PI); hours/wk spent in PA | 3m; delivered by trained YMCA staff; theory: none | Children attended three 1hr sessions/wk; families attended once per week to encourage total participation. Sessions consisted of organised PA (e.g. soccer, dancing, swimming) at the YMCA, with reduced joining fees at the program close. Educational curriculum (10 1hr sessions/week) for parents (based on Eat Smart Move More) was also delivered, including strategies for improving PA and limiting SV. No control group. | **Favoured intervention**  Hrs/wk spent in PA significantly increased at 3m and 12m (Cohen’s d = 3.55; 95% CI =1.00, 6.10, p= 0.01) and (Cohen’s d = 3.6; 95% CI = 1.2, 6.1, p<0.01). |
| **MEND 7-13**(18)  Baker; 2012; UK  Narrative review, realist synthesis | Trial (one group, pre- and post-measures); N/A | N=212 families; not reported  Age: 10.5 ± 1.8  % male: not reported  Weight status: eligibility criteria required overweight status (defined by BMI >91st percentile) | Baseline, 10wk; measure not reported; hrs and days participating in PA/week | 10wk; delivered by local staff from a variety of backgrounds (including healthcare, PA and other professionals); theory: none | 20 2hr group sessions held twice/wk including behaviour change, nutrition and PA education. No control group. | **Mixed evidence**  Mean days participating in PA/wk 1.2 (not significantly different from 1.3 national average); mean hours participating in PA/week 3.2 (compared with 3.8 national average, p=0.0001). |
| **No name reported**(19)  Bacardi-Gascon; 2012; Mexico  Narrative review only | CRCT; schools | N=478; 90%  Age: 8.5 ± 0.7  % male: 51  Weight status: BMI 18.5 ± 3.7 | Baseline, 6m, 9m, 15m, 24m; self-reported (INTA PA questionnaire); hr/day in supervised sports or dance, PE, and outdoor play | Classroom curricula 8wk and parent education 4m; delivered by trained nutrition and PA counsellors, plus nutrition graduate students; theory: Bronfenbrenner's Ecological Model | School board/teachers received three 60min sessions discussing healthy lifestyles and how to improve school environment for diet and PA, children received one 30min/wk interactive lesson to encourage healthy eating and increased PA, and parents received 60min/m nutrition and PA education session. Control schools treatment not reported. | **Mixed evidence**  Intervention group reported increase at baseline in supervised sports or dance (1.35 SD 2.01 vs. 2.12 SD 2.49 hours/wk, p=0.0001) and PE (0.90 SD 0.39 vs. 0.97 SD 0.15 hours/week, p=0.0003) compared to control, but a decrease in outdoor playing (1.42 SD 0.91 vs. 1.39 SD 0.85 hours/day, p=0.63). |
| **No name reported**(20)  Centis; 2012; Italy  Narrative review, realist synthesis | RCT; families | N=198; not reported  Age: 9.3 ± 0.3  % male: 48  Weight status: BMI 18.2 ± 2.8 | Baseline, 8m; self-reported (interview); time spent in outdoor activities and extracurricular sports | 8m; delivered by trained counsellors, physicians and graduate students; theory: none | Three fortnightly meetings were held with children to encourage PA and distribute pedometers for self-tracking of improved steps. Additional nutrition meeting in which children were educated in healthy food choices. Parents encouraged to attend three motivational meetings and received weekly phone calls to inform, sensitise and motivate them to further improve their children's lifestyle. Control group treatment not reported. | **Favoured intervention**  Increase in outdoor activities for intervention (6.23 to 9.93 hours/week, p<0.001), compared with relatively small increase in control (6.28 to 7.21, p=0.279). Time spent in extracurricular sports remained stable in intervention group (-0.12 hour/week, p=0.152), but was reduced in control (-0.35 hour/week, p=0.003). |
| **ABC Study (internet version of Chen, 2010**(21)**)** (22)  Chen; 2011; USA  Narrative review, meta-analysis, realist synthesis | RCT; children | N=50; 79%  Age: 12.5 ± 1.5  % male: 54 Weight status: approx. 35% overweight (defined by BMI >85^th^ percentile) | Baseline, 2m, 6m, 8m; objective (ActiGraph MTI/CSA 7164 accel); accel count | 2m; delivered online; theory: Transtheoretical Model of Behaviour Change (information tailored to stage of change) and Social Cognitive Theory | Internet content consisted of activities to enhance self-efficacy of adolescents and facilitated their understanding and use of problem-solving skills related to nutrition, PA and coping. Participants also received a pedometer with instructions for recording steps online. Parents received three 15min sessions of online content, designed to increase parents' knowledge and skills regarding dietary intake and adolescent’s PA. Participants in the control group logged on to website and received non-tailored general health information. | **Favoured intervention**  Actigraph count difference between groups over time: 12.46 (group x time effect estimate), p=0.001, 95%CI 6.62, 18.41 (p<0.05 significant change for baseline-2m, baseline-6m, baseline-8m for PA in intervention group). |
| **The Family Project**(23)  Coppins; 2011; UK  Narrative review only | RCT; children | N=46; 71%  Age: 10.3 (SD not reported)  % male: 34 Weight status: BMI 27.5 (SD not reported) | Baseline, 6m, 12m, 18m, 24m; mixed (7-day PA diary and Yamax Digi Walker pedometer); steps/day, MVPA min/wk | 12m; delivered by a dietician, PA health promotion officer, and educational/clinical psychologist and 2-3 PA instructors; theory: none | Children attended 2 4hr workshops (held 1-2 wks apart), plus PA sessions (1hour/week) during term time over the one year intervention. Siblings and family members encouraged to participate. Workshops focused on healthy eating, PA, reducing SB, behaviour change and psychological well-being. PA sessions included junior gym sessions (bikes and weights), circuits, trampolining, rock climbing etc. Cross-over control design ensured participants received the intervention 12m after baseline. | **Did not favour intervention**  No difference between groups for mean pedometer steps/day (baseline or any follow-up), nor for low or high intensity activity. Total minutes of MPA undertaken per week was significantly greater for the C/I group at 24m (i.e. just after they had finished the program), I/C mean 182.9min (95%CI -39.2, 404.9); C/I mean 606.9 min (95%CI 202.7, 1.1.10) p=0.038. |
| **Healthy Homework**(26)  Duncan; 2011; New Zealand  Narrative review, realist synthesis | CRCT; classes | N=97; not reported  Age: not reported  % male: 36 Weight status: not reported | Baseline, 6wk; objective (undisclosed sealed pedometer); steps/day | 6wk; delivered by teachers and online; theory: Information-Motivation-Behavioural Skills model, Theory of Reasoned Action, Theory of Planned Behaviour, Social Cognitive Theory, Control Theory, Operant Conditioning | Each child received a homework booklet organised into five PA and five nutrition topics: 1 walking/fruit and vegetables, 2 television/breakfast, 3 sports/drinks, 4 fun games/food shopping, 5 fitness/cooking. PA tasks required with each topic, including family walks around the neighbourhood, walking to and from school, limiting TV, coaching parents in a particular sport, inventing a fun game (individual or team), testing the fitness of the family, swimming at local pool (subsidised entry). Colourful wristbands provided for those meeting task requirements. Family resources and equipment provided (plus in-class material to support intervention for teachers). The control group received no intervention. | **Favoured intervention**  Absolute steps non-significantly declined in control group and non-significantly increased in intervention group. Mean step count in the control group declined (10990 to 9510) during intervention period, whilst intervention group increased (10350 to 11480). No intervention effect observed for active transport or sports participation. Significant intervention effect of 2830 more steps/day (95%CI 560, 5300; p=0.013). Independent sex effect - boys averaging 2500 more steps/day than girls (p=0.005). |
| **One Body One Life**(27)  Towey; 2011: UK  Narrative review only | Trial (one group, pre- and post-measures); N/A | N=186; 41%  Age: not reported  % male: 50 Weight status: 14% overweight and 19% obese | Baseline, 10-12wk; self-reported (PA recall); frequency of PA during past week | 10-12wk; delivery not reported; theory: none | Sessions consist of a healthy eating and a PA workshop (held with adults and children together), to assist participants in making healthy choices. A 45min PA workshop followed; main objective was the development of core motor skills, confidence, and self-esteem alongside improving fitness. No control group. | **Favoured intervention**  Average change in activity units of more than 60 minutes 3.9 per week (95%CI 2.5, 5.2, p<0.001). |
| **Reach Out**(28)  Burnet; 2011; USA  Narrative review, realist synthesis | Trial (one group, pre- and post-measures); N/A | N=24; 39%  Age: 10.9 ± 1.2  % male: 27 Weight status: BMI 33.7 ± 6.1 | Baseline, 4m, 12m; self-reported (questionnaire not named); time spent in activity of vigorous and light intensity activity, and time spent walking | 14wk; designed by a behavioural scientist and paediatrician, who trained lay community leaders to deliver; theory: none | Program included behavioural goal setting and self-monitoring; skills building; group problem solving; and engaging family activities, alternating weekly focus between nutrition and PA. No control group. | **Favoured intervention**  Percentage completing more than 20min vigorous exercise/3 days in past week; 46% baseline, 58% 4m, 39% 12m (p=0.5). Percentage completing more than 20min light exercise/3 days in past week; 39% baseline, 50% 4m, 61% 12m (p=0.34). Percentage completing more than 2hours walking/day; 26% baseline, 58% 4m, 56% 12m (p=0.07). |
| **TEAM**(29)  Greening; 2011; USA  Narrative review, realist synthesis | CRCT; schools | N=450; 100%  Age: 8.3 ± 1.3  % male: 52  Weight status: approx. 33% overweight (defined by BMI >95^th^ percentile) | Baseline, 8m; self-reported (21-item PA checklist); frequency of PA | 8m; delivery not reported; theory: none | Monthly events (alternating between nutrition and physical activities/contests) e.g. healthy tailgating, parent-child softball throw contest - designed to coincide with community events. Cooking and PA equipment distributed as prizes. The control group followed the state's standard health curriculum, which included didactic nutrition education, health information incorporated into academic lessons, and weekly PE classes. | **Favoured intervention**  Intervention school reported engaging in significantly more PA from baseline to post-intervention whereas the control school reported a decline, range of possible PA scores 0-21. Intervention group pre 6.24, post 7.00. Control group pre 7.04, post 6.49. F(1, 449) = 4.56, p=0.04. |
| **Triple P**(30)  Golley; 2011; Australia  Narrative review, realist synthesis | RCT; children | N=54; 49%  Age: 8.2 ± 1.1  % male: 37 Weight status: 74% obese (reported as defined by international cut-points) | Baseline, 6m, 12m; parent-reported (20-item PA checklist); time spent in ‘active play’ | 8wk (plus 7 sessions over undisclosed period); delivered by first author who developed the lifestyle education component and undertook accredited training for the parenting component; theory: none | Two intervention arms (plus wait-list control): PA group, and PA+Diet group. All parents attended standardised general parenting program comprising 4/wk 2hr group session and 4/fortnight 20min telephone sessions. Parents in PA+Diet also participated in additional seven lifestyle education sessions based on the Australian food selection guide as well as SB and PA recommendations (children attended structured, supervised PA sessions during these periods). Cross-over control design ensured participants received the intervention 12m after baseline, plus a generic ‘lifestyle’ pamphlet. | **Did not favour intervention**  There were no differences at any time point by study group. Regardless of study group there was a significant increase in the time reported spent in active play. Baseline active play 230 (145-367) min/day, 6m250 (160-414) minday, 12m288 (166-423) min/day, p=0.06. |
| **BOUNCE** **(weekly)**(31)  Olvera; 2010: USA  Narrative review only | Trial (two groups, pre- and post-measures); N/A | N=35 (mother-daughter dyads); 95%  Age: 10.2 ± 1.1  % male: girls only  Weight status: 66% overweight | Baseline, 3m; objective (ActiCal accel); MVPA minutes and accel counts/min | 3m; delivered by child psychologist and licensed counsellor, registered dietician/nutrition educators, and trained Cooper Institute fitness specialists; theory: none | Three 90min group sessions/wk, consisting of structured 'aerobic or sport' sessions, or free play recreational activities, nutrition sessions, and behavioural counselling sessions. Comparison group met with instructor once a week; received written educational materials on various nutrition/ behavioural counselling topics and light intensity exercise. | **Did not favour intervention**  No significant difference in MVPA in either group. Effect size for change in MVPA (Cohen's d=0.75; p=0.049: EG 70.7 (31.5) CG 38.0 (13.1)). Within group analyses, if conducted, was not reported. |
| **BOUNCE** **(daily)**(32)  Olvera; 2010: USA  Narrative review only | Trial (one group, pre- and post-measures); N/A | N=37 (mother-daughter dyads); 80%  Age: 10.8 ± 1.2  % male: girls only  Weight status: BMI 29.2 ± 6.6 | Baseline, 1wk, 2wk, 3wk; objective (ActiCal accel); MVPA minutes and accel counts/min | 3wk; delivered by child psychologist and licensed counsellor, registered dietician/nutrition educators, and trained Cooper Institute fitness specialists; theory: Social Cognitive Theory | Daughters attended daily sessions (9am-5pm) of exercise (fun and varied opportunities to be active, and discussed PA benefits, components of a healthy lifestyle, strategies to over-coming barriers etc.), nutrition education, and behavioural counselling. Parents participated in one 2hr session/wk with a dietician, fitness instructor and counsellor to teach them how to adapt family meals, and activities to support to daughters' healthy choices and enhance their PA and self-esteem. No control group. | **Favoured intervention**  Daughters’ mean MVPA improved across each week of the intervention (as well as over the entire duration). End of Wk 1: 60.47min, Wk 2: 70.32min (compared to wk1 t=-3.60, p<0.01), Wk 3: 74.70min (compared to wk2 t=-2.89, p<0.05). Wk 3 compared with wk1 t=-6.55, p<0.01). |
| **HIKCUPS**(33)  Cliff; 2010; Australia  Narrative review, realist synthesis | RCompT; children | N=87; 53%  Age:8.2 ± 1.1 % male: 41 Weight status: 78% obese (remaining sample were overweight) | Baseline, 6m, 12m; ActiGraph 7164 accel; total PA (counts/min), % time spent in MPA, VPA, and MVPA | 10wk contact phase followed by 3m maintenance phase; delivered by two qualified PE teachers; theory: Competence Motivation Theory | Participants attended a 2hr weekly contact session, followed by a minimal contact maintenance phase that promoted PA and reduced time spent in recreational SB by fostering movement skill proficiency and enhancing perceptions of physical competence (included 90min developmental activity/session). Home challenges (with rewards) were set, and parents attended a workshop covering behaviour monitoring, barrier identification, problem-solving, planning and goal-setting. Control group participants received a dietary modification program (parent-only). | **Did not favour intervention**  Non-significant group-by-time effects for all physical activity outcomes (all p>0.05). |
| **No name reported**(34)  Rhodes; 2010; Canada  Narrative review, realist synthesis | *Pilot* RCT; families | N=65 families; 61%  Age: not reported  % male: not reported  Weight status: not reported | Baseline, 4wk; GLTPAQ, IPAQ and BRFSSS used to create composite measure; total minutes and frequency of bouts of family PA | 4wk; delivered by mail; theory: theories of intention and regulatory behaviour | Families received information package consisting of PA guidelines, local municipal healthy/active living guide and planning material (encouraged to plan for ‘when’, ‘where’, ‘how’ and ‘what’ PA using family calendar and workbook provided). Materials also focused on problem solving barriers to PA. Control group participants received information package consisting of PA guidelines, and local municipal healthy/active living guide, but not planning material. | **Favoured intervention**  The intervention resulted in significant time effects for all parent-reported family PA measures; these results favoured increases in PA from baseline to 4wk post-test (F = 4.23 to 17.63; g2 = 0.06 to .22). The intervention group, reported significantly higher informal/unstructured PA frequency (F = 7.31; g2 = .11) and total minutes (F = 6.49; g2 = .09) which translated into significantly higher total family PA frequency (F = 5.31; g2 = .08) and total minutes (F = 4.26; g2 = .06) compared to the standard condition in time x group interactions. |
| **ABC Study**(21)  Chen; 2010; USA  Narrative review, realist synthesis | RCT; families | N=67; 93%  Age: 9.0 ± 0.9  % male: 57  Weight status: BMI 19.2 ± 3.1 | Baseline, 2m, 6m, 8m; objective (Caltrac PA computer); accel count | 2m; delivered by trained bilingual counsellors and registered dieticians; theory: Social Cognitive Theory | Children participated in one 45min session/wk, consisting of play-based education to increase self-efficacy and facilitate understanding of PA, nutrition and coping behaviours. Sessions also included 15min PA, plus receipt of a pedometer. Parents attended two 2hr sessions to increase parent's knowledge of/support for PA and dietary intake by discussing techniques and distributing workbooks. Cross-over control design ensured participants received the intervention 6m after baseline. | **Favoured intervention**  Caltrac count difference between intervention and control groups: 428.7, p=0.001, 95%CI 236.1, 621.3 (additionally, p<0.05 significant change for baseline-2m, baseline-6m, baseline-6m for PA in intervention group). |
| **Fit ‘n’ Fun Dudes**(35)  Hardman; 2009; UK  Narrative review only | CRCT; schools | N=29; not reported  Age: 10.6 ± 0.8  % male: girls only  Weight status: BMI 19.9 ± 3.7 | Baseline, 'during 8-day intervention phase', 3m; objective (Yamax SW-200 pedometer); PA, steps/day | 8 days; delivered in family home (facilitator details not reported); theory: none | Children given educational and motivational materials 'from' the Fit 'n' Fun Dudes, plus step count targets to receive healthy daily rewards (provided to parents). Progress recorded on step count chart. Maintenance letters distributed for 12wks following intervention. The control group received no intervention. | **Favoured intervention**  During intervention (weekdays), experimental children significantly more active than control children. Non-significant trend maintained at follow-up period. On weekend days, experimental children more active than control children during intervention and at follow-up, mean weekday increase 4112 steps/day during intervention (t=-2.96, p=0.006); mean weekend increase 5318 steps/day during intervention (t=-3.57, p=0.003) and at follow-up (t=-2.86, p=0.01. Significant time x group interaction 7.42; p=0.001. |
| **Family Connections**(36)  Estabrooks; 2009; USA  Narrative review, realist synthesis | RCompT; families | N=170; 26%  Age: 10.7 (no SD reported)  % male: 54  Weight status: BMI 27.2 (SD not reported) | Baseline, 6m, 12m; self-reported (BRFSSS); MPA days/wk, VPA days/wk | FC-groups (2 wk), FC-workbook (1wk), FC-IVR (10 telephone sessions delivered over unspecified duration); delivered by a study research assistant and a dietician; theory: Socio-Ecologic Theory | FC group consisted of two-small group sessions (2hr each, spaced 1wk apart) held at local clinic, focussing on parent's behavioural health skills and knowledge of weight, nutrition and PA. It also identified key parenting skills: limit setting, effective communication, and role modelling, problem solving, development of action plans etc. FC-W group received a workbook promoting PA and other healthy behaviours. FC-IVR group parents completed the two-session FC small-group program, and were subsequently provided with 10 follow-up sessions delivered via IVR (interactive voice technology). Cross-over control design ensured participants received the intervention 6m after baseline. | **Favoured intervention**  Participants in FC-IVR reported a significant increase in the number of days they participated in VPA from baseline (mean 3.75 SD 2.43) to 6m baseline (mean 3.75 SD 2.43), and from baseline to 12m (mean 3.47 SD 2.09). Similar results across MPA. |
| **No name reported**(37)  Hovell; 2009; USA  Narrative review, realist synthesis | RCT; families | N=117 families; 85%  Age: 11.5 ± 1.0  % male: 42 Weight status: not reported | Baseline, 3m, 9m, 12m; self-reported (24 hour PA recall); high impact PA | 8 wks; delivered by bilingual (English and Spanish) instructors; theory: Learning Theory. | Parents received 90min/wk instruction on how to encourage PA and diet change in children; discussion, activities and exercises to take home. Behaviour management techniques also taught; reinforcement, shaping, modelling, monitoring, goal setting, and behaviour contracting to improve target behaviour. Children received 90 min/wk - of which at least 60min were supervisory PA - remainder of time spent setting and reviewing individual PA goals. Families also received brief coaching calls throughout intervention period. Comparison group received identical duration/format of intervention, but with injury prevention content. | **Mixed evidence**  A significantly higher % of experimental boys met PA recommendations at 3m compared to control boys (not 9m or 12m; experimental boys % declined at 9m and 12m). Experimental girls reported higher % of meeting PA recommendations at all time points (not statistically significant). No group by time effects for high impact physical activity across the length of the study, pre-test to post-test or post-test to follow-up for the full sample or either sex separately. |
| **Shining like stars**(38)  Trost; 2009: USA  Narrative review, meta-analysis | CRCT; churches | N=95 (parent-child dyads); 90%  Age: 8.9 ± 1.8  % male: 48 Weight status: not reported | Baseline, 1wk, 2wk, 3wk, 4wk’; parent-reported (questionnaire developed by authors); number of days with at least 60 min MVPA | 4wk; delivered by volunteer Sunday School teachers and children’s ministry co-ordinators at each church; theory: none | Sunday Schools implemented a 4-lesson curricula focussing on providing learning experiences (with religious theme) and incorporating opportunities to perform MVPA. Families also conducted 3 devotional physical activities (outside of Sunday School environment), reinforcing parent's role and bringing family together (e.g. local park visits). Control group received religious curricula, without incorporated PA. | **Did not favour intervention**  No significant difference for PA outside of Sunday classes, but significant treatment x time interaction for screen time.  Those attending the intervention churches declined from 1.5 (95%CI 1.3-1.7) hours/day to 1.2 (95%CI 1.1-1.5) hr/day. Mean difference 0.2 hr/day (95%CI 0.04-0.4). Daily screen time in control churches remained constant at 1.4 (95%CI 1.1-1.6) at baseline to 1.4 (95%CI 1.2-1.7) at completion. |
| **No name reported**(39)  Chen; 2008; USA  Narrative review, meta-analysis, realist synthesis | Trial (one group, pre- and post-measures); N/A | N=42; 21%  Age: 8.8 ± 0.09  % male: 69  Weight status: BMI 18.3 ± 3.8 | Baseline, 1m, 6m; parent-reported (FEAHQ); frequency with which the parent, spouse, and child engage in PA and SB | 2wk; phone consultation delivered by ‘researchers’ (no further details provided); theory: Ecological Model of Childhood Obesity Prevention. | Mothers provided with individually tailored educational materials on nutrition, PA and health weight maintenance based on baseline assessment, follow-up phone consultation to answer any questions. No control group. | **Favoured intervention**  PA time effect estimate 2.60 (95% CI 0.46, 4.75, p=0.02). |
| **Share-AP Action**(40)  Anand; 2007; Canada  Narrative review, realist synthesis | RCT; families | N=159; 91%  Age: 10.4 ± 3.1  % male: 39  Weight status: BMI 21.7 ± 4.8 (5-10 yr) and BMI 25 ± 5.8 (11-18 yr) | Baseline, 6m; self-reported (24-hr PA recall); proportion of time spent in PA of different intensity | 6m; delivered by a trained Aboriginal health counsellor; theory: Protection Motivation Theory, normative influences and Theories of Persuasion. | ‘Regular’ home visits to assess and set dietary and PA goals for each household member, and the family as a whole. The control group received Canadian national guidelines on diet and PA. | **Favoured intervention**  Intervention group reported 3.6% rise in proportion highly active (21.7 to 25.3), compared with 6.3% decrease in control (28.1 to 21.8); p=0.35. |
| **No name reported**(41)  Shelton; 2007: Australia  Narrative review, meta-analysis | Wait-list RCT; families | N=43; not reported  Age: 7.6 ± 2.0  % male: 45  Weight status: BMI 26.6 ± 2.7 | Baseline, 3m; self-reported (3-day PA diary); PA hrs/day | 1m; delivered by the "relevant professionals" including paediatrician, dietician, physiotherapist, psychologist; theory: none | Initial assessment session attended by parent and child, followed by 4 weekly 2hr parent-only sessions, including a range of adult education and facilitation techniques (visual aids, hand-outs, brainstorm exercises, practical demonstrations, worksheets, guided discussions) to encourage active participation and group cohesion. Focused on overweight/obesity 'knowledge' tools, activity, nutrition and motivation. Each family member also received a parent manual. Control group treatment not reported. | **Did not favour intervention**  There were no differences between treatment and control for PA measure in children. Control group baseline mean hours/day 2.23 (1.05), 3m 2.05 (0.74). Treatment baseline 2.20 (0.61), 3m 2.19 (0.62). |
| **No name reported**(42)  Rodearmel; 2006; USA  Narrative review only | RCT; families | N=88 families 77%  Age: 11.1 ± 0.5  % male: 53 Weight status: mean BMI percentile 94% | Baseline, 1-14wks continuous; objective (Accusplit AE120 pedometer); steps/day | 14wk; delivery not reported; theory: none | Participants given pedometers and asked to gradually increase their steps by at least 2000 steps/day above their individual baseline level. Participants were given 'fun, creative, family-oriented, educational logs’ to record progress. All participants met with study staff three times during study to report on progress, collect data and encourage continued participation. Control group received and logged pedometer steps but received no advice. | **Favoured intervention**  Steps/day increased in all members of intervention families - but not in any control families. Intervention target girls steps/day: wk1: 8347 to wk2: 14 10054 (p<0.0001 vs baseline and p<0.05 vs control), Intervention target boys: 9553 to 11482 (p<0.0001 vs baseline and p<0.05 vs control). |
| **Growing Healthy Families**(43)  Rooney; 2005: USA  Narrative review only | RCT; families | N=316; 90%  Age: 9.7 (no SD reported)  % male: 49  Weight status: mean BMI percentile 82.2 (SD not reported) | Baseline, 3m, 9m; self-reported (questionnaire not reported); time spent playing and in SB | 3m; delivery not reported; theory: none | Families randomised to either pedometer + education (PE), pedometer only (P), or control group (C). PE and P given pedometer, instructed to walk 10,000 steps daily for intervention period and record step counts in log, plus bi-weekly newsletter that provided fun educational tips for increasing activity. PE families also attended six 1hr bi-weekly sessions concerning nutrition, PA and other 'parenting' issues. All families completing follow-up measures received $25 stipend, and were eligible to win a $4000 trip to Walt Disney World. The control group received no intervention. | **Favoured intervention**  All PE participants (parents and children) reported significant improvements in normative exercise assessment (compared with P and C groups) at 3m, but change not significant at follow-up. Overall,15% of parents reported spending more time playing with their children post-program, and 10% nine months later, but these changes were not significant (p=0.6476, and 0.9124, respectively). |
| **GET FIT**(44)  Ransdell; 2004: USA  Narrative review, meta-analysis, realist synthesis | RCT; granddaughter-mother-daughter triads | N=13; 25%  Age: 10.1 ± 1.5  % male: girls only  Weight status: not reported1 | Baseline, 6m; mixed (Youth BRFSSS, and Yamax SW701 pedometer); steps/day, frequency aerobic activity | 6m; delivery not reported; theory: none | Families given a home-based packet of information during two 2hr classroom sessions - including calendar of recommended activities, photos of various exercise activities, schedule for mailing logs, pedometers. Asked to complete structured bouts of exercise 3 times/wk (aerobic activity, flexibility, and strength training suggested). Triads encouraged, but not required, to exercise together. The control group received no intervention, but participants told they would receive intervention materials after the study period. | **Favoured intervention**  Large improvement for aerobic and strength-building activities (r=0.145-0.167; 107-209% intervention to 0.37-20% control), but not significant. Participation in flexibility significantly improved for intervention groups by 305%, compared with 15% reduction for control group. Steps/day significantly improved for intervention group (+37% compared with -13% for control). Group x time interaction significant for pedometer steps, flexibility; effect size = 0.443, F-value = 20.68, p<0.001. Steps/day; effect size = 0.284, F-value = 13.86, p=0.001. |
| **GEMS-Memphis**(45)  Beech; 2003: USA  Narrative review, realist synthesis | Trial (two groups, pre- and post-measures); parent-daughter dyads | N=60; 100%  Age: 8.9 ± 0.8  % male: girls only  Weight status: BMI 23.7 ± 6.3 | Baseline, 3m; objective (CSA accel); MVPA min/day and count/min | 13wk; delivered by a trained graduate student, and a member of the local community centre staff trained as a lay health educator; theory: Social Cognitive Theory | Parent-daughter pairs divided into one of three conditions; child-targeted, parent-focused and comparison group. Child-targeted: weekly, 90min intervention sessions divided into a PA component (e.g. dance session) and a nutrition component, focusing on knowledge and behaviour change skills to promote healthy eating and increased PA. Parent-targeted: weekly, 90min sessions with similar content reinforcing key points and providing take-home materials for parents only (children attended care during session). Parents also participated in short dance segments. Control group was an ‘active placebo', received non-nutritional/PA information to promote positive self-esteem and cultural enrichment. | **Favoured intervention**  Relative to the comparison group, the active interventions (when averaged) demonstrated an 11.7% increase in MVPA min/day, accel count/min child-targeted group: 361.0 (17.3), parent-targeted group: 387.9 (17.2), comparison: 347.3 (18.2). Adjusted mean difference -18.0 (SE 24.4, p=0.45). Min MVPA child-targeted group: 72.0 (8.2), parent-targeted group: 78.8 (8.2), comparison: 67.5 (8.5). Adjusted mean difference: -6.8 (SE 11.7, p=0.54). |
| **GEMS-Minnesota; substudy called 'Girlfriends for Keeps'** **(Keys to Eating, Exercising, Playing and Sharing)**(46)  Story; 2003: USA  Narrative review, meta-analysis | *Pilot* RCT; children | N=53; not reported  Age: 9.3 ± 0.9  % male: girls only  Weight status: BMI 20.7 ± 4.9 | Baseline, 3m; objective (CSA accelerometer); counts/min and time spent in MVPA | 3m; delivered by trained African American instructors; theory: Social Cognitive Theory | After-school program held twice/wk (1hr sessions), focused on increasing MVPA and decreasing sedentary time, and experiencing feelings of enjoyment, physical competence, self-confidence in performing PA (plus dietary goals). Meetings consisted of fun, culturally appropriate, interactive, hands-on activities and education to emphasise skill-building and practice health behaviours. Incentives provided for attendance, and setting and achieving short-term goals. All reinforced by family activities, including weekly information packets, encouraging phone calls, family night, and organised neighbourhood walks. Pedometers provided to encourage and chart daily PA. Control group was an ‘active placebo', received non-nutritional/PA information to promote positive self-esteem and cultural enrichment. | **Favoured intervention**  PA consistently greater levels for intervention group at follow-up: accel counts/min (mean difference 57.4 (36.5)), MVPA (mean difference 2.9 (13.7)) and self-report PA GAQ met-adjusted usually score (0.3 (0.5)). |
| **The Family Health Project**(47)  Nader; 1992; USA  Narrative review only | CRCT; schools | N=602; not reported  Age: 12.0 ± 1.7  % male: 52 Weight status: not reported | Baseline, 3m, 12m, 24m, 36m, 48m; self-reported (7-day PA recall); time spent in PA | 12m; delivered by facilitators who were extensively trained and supervised weekly; theory: Social Learning Theory | Intervention split into 12wks of intensive education, followed by six maintenance sessions over a 9m period. Ethnically homogenous groups attended 90min evening sessions of training in self-monitoring, goal-setting, problem-solving, self-rewarding goal achievement, and peer support. Sessions included aerobic exercise, behaviour management, education and healthy snacks. Control group treatment not reported. | **Did not favour intervention**  Significant intervention effects were only reported for one observation (Anglo boys at 48m). ANOVA results (compared to baseline): +2.23 (SE 1.10) KKD (p=0.021). NO other intervention effects were reported. |
| **The Family Health Project**(48)  Patterson; 1988: USA  Narrative review, meta-analysis | RCT; families | N=30 (families); not reported  Age: not reported  % male: not reported Weight status: not reported | 1hr; objective (direct observation whilst at family zoo); number of metres travelled, proportion of intervals active, and proportion using escalator during observation | 12m; resources developed by a team of psychologists, health educators, dieticians and exercise physiologists, but intervention delivery not reported; theory: Social Learning Theory | Intervention split into 12wks of intensive education, followed by six maintenance sessions over a 9m period. Ethnically homogenous groups attended 90min evening sessions of training in self-monitoring, goal-setting, problem-solving, self-rewarding goal achievement, and peer support. Sessions included aerobic exercise, behaviour management, education and healthy snacks. Control group treatment not reported. | **Mixed evidence**  Mexican-American intervention families walked significantly farther/spent more time active than controls. There were no statistically significant differences on either of these variables between intervention and control Anglo families, Mexican-American families only: travelled 1868.7 metres during hour (SD 625.0) compared with control 1512.5 (SD 484.4) - t=7.6 (p<0.01). Proportion of intervals active (walking, or very active) 0.57, compared with 0.50 control (x2 = 4.9, p<0.05). Proportion using escalator 0.29, compared with 0.58 (x2 = 9.6, p<0.01). |
| **The Family Health Project**(49)  Nader; 1983: USA  Narrative review only | RCT; families | N=78; not reported  Age not reported  % male: not reported Weight status: not reported | Baseline, 12wk; self-reported (questionnaire not reported); time spent in PA | 8wk; delivered by group facilitators (study authors); theory: Cognitive-Social Learning theory | Ethnically homogenous groups attended 90min evening sessions of training in self-monitoring, goal-setting, problem-solving, self-rewarding goal achievement, and peer support. Sessions included aerobic exercise, behaviour management, education and healthy snacks. Control families received same extrinsic reward system, data collection instruments and printed materials, but did not attend weekly sessions. | **Did not favour intervention**  ANOVA-utilising treatment condition, age, and ethnicity shown no main effects in reported minutes of exercise; no other details provided. |

Accel: accelerometer, BMI: body mass index, BRFSSS: Behaviour Risk Factor Surveillance Survey System, C/I: control then intervention, CB: community-based, CG: control group, CRCT: cluster randomised controlled trial, EG: experimental group, FC: Family Connections, FC-W: Family Connections Workbook group, FC-IVR: Family Connections Interactive Voice Recording group, FEAHQ: The Family Eating and Activity Habits Questionnaire, GLTPAQ: Godin Leisure Time Physical Activity Questionnaire, HLBS: Healthy Lifestyle Behaviour Scale, HB: home-based, hr: hours, I/C: intervention then control, INTA: Institute of Nutrition and Food Technology (at the University of Chile), IPAQ: International Physical Activity Questionnaire, IVR: interactive voice recording, KKD: kilocalories per kilogram body weight per day, min: minutes, MET: metabolic equivalent, m: months, MPA: moderate physical activity, MVPA: moderate-to-vigorous physical activity N/A: not applicable, PA: physical activity, PE: physical education, PI: principal investigator, RCT: randomised controlled trial, RCompT: randomised comparison trial, SB: sedentary behaviour, SD: standard deviation, SES: socio-economic status, ST: sitting time, SV: screen viewing, VPA: vigorous physical activity, wk: weeks, UK: United Kingdom, USA: United States of America, YMCA: Young Men’s Christian Association, yr: years

1. Nyberg G, Sundblom E, Norman Å, Bohman B, Hagberg J, Elinder LS. Effectiveness of a Universal Parental Support Programme to Promote Healthy Dietary Habits and Physical Activity and to Prevent Overweight and Obesity in 6-Year-Old Children: The Healthy School Start Study, a Cluster-Randomised Controlled Trial. PLoS One [Internet]. Jan [cited 2015 Feb 16];10(2):e0116876. Available from: http://www.ncbi.nlm.nih.gov/pubmed/25680096

2. Arredondo EM, Morello M, Holub C, Haughton J. Feasibility and preliminary findings of a church-based mother-daughter pilot study promoting physical activity among young Latinas. Fam Community Health [Internet]. 2014;37(1):6–18. Available from: http://www.ncbi.nlm.nih.gov/pubmed/24297004

3. Newton RLJ, Marker AM, Allen HR, Machtmes R, Han H, Johnson WD, et al. Parent-Targeted Mobile Phone Intervention to Increase Physical Activity in Sedentary Children: Randomized Pilot Trial. J Med INTERNET Res [Internet]. Canada; 2014 Nov 10 [cited 2014 Nov 11];16(11):e48. Available from: http://www.ncbi.nlm.nih.gov/pubmed/25386899

4. De Bock F, Genser B, Raat H, Fischer JE, Renz-Polster H. A participatory physical activity intervention in preschools: a cluster randomized controlled trial. Am J Prev Med. 2013/06/26 ed. 2013;45(1):64–74.

5. De Bock F, Fischer JE, Hoffmann K, Renz-Polster H. A participatory parent-focused intervention promoting physical activity in preschools: design of a cluster-randomized trial. BMC Public Health [Internet]. 2010 Jan;10:49. Available from: http://www.pubmedcentral.nih.gov/articlerender.fcgi?artid=2835684&tool=pmcentrez&rendertype=abstract

6. Eather N, Morgan PJ, Lubans DR. Feasibility and preliminary efficacy of the Fit4Fun intervention for improving physical fitness in a sample of primary school children: a pilot study. Phys Educ Sport Pedagog. 2013 Sep;18(4):389–411.

7. Morrison R, Reilly JJ, Penpraze V, Westgarth C, Ward DS, Mutrie N, et al. Children, parents and pets exercising together (CPET): exploratory randomised controlled trial. BMC Public Health. England; 2013;13:1096.

8. Smith LR, Chadwick P, Radley D, Kolotourou M, Gammon CS, Rosborough J, et al. Assessing the short-term outcomes of a community-based intervention for overweight and obese children: The MEND 5-7 programme. BMJ Open. 2013/05/07 ed. 2013;3(5).

9. Catenacci V, Barrett C. Changes in physical activity and sedentary behavior in a randomized trial of an internet-based versus workbook-based family intervention study. J Phys Act Heal [Internet]. 2014 [cited 2014 Nov 24]; Available from: http://europepmc.org/abstract/med/23364318

10. Siwik V, Kutob R, Ritenbaugh C, Cruz L, Senf J, Aickin M, et al. Intervention in overweight children improves body mass index (BMI) and physical activity. J Am Board Fam Med. 2013/03/09 ed. 2013;26(2):126–37.

11. Barr-Anderson D, Adams-Wynn A, Alhassan S, Whitt-Glover M. Culturally-appropriate, Family- and Community-based Physical Activity and Healthy Eating Intervention for African-American Middle School-aged Girls: A Feasibility Pilot [Internet]. J Adolesc Fam Health. 2014 [cited 2015 Jan 9]. Available from: http://scholar.utc.edu/jafh/vol6/iss2/6

12. Delamater AM, Pulgaron ER, Rarback S, Hernandez J, Carrillo A, Christiansen S, et al. Web-based family intervention for overweight children: a pilot study. Child Obes. 2013/01/12 ed. 2013;9(1):57–63.

13. Jago R, Sebire SJ, Turner KM, Bentley GF, Goodred JK, Fox KR, et al. Feasibility trial evaluation of a physical activity and screen-viewing course for parents of 6 to 8 year-old children: Teamplay. Int J Behav Nutr Phys Act [Internet]. 2013/03/21 ed. 2013 Jan;10:31. Available from: http://www.ncbi.nlm.nih.gov/pubmed/23510646

14. Finkelstein EA, Tan YT, Malhotra R, Lee CF, Goh SS, Saw SM. A Cluster Randomized Controlled Trial of an Incentive-Based Outdoor Physical Activity Program. J Pediatr. 2013/02/19 ed. 2013;

15. Crespo NC, Elder JP, Ayala GX, Slymen DJ, Campbell NR, Sallis JF, et al. Results of a multi-level intervention to prevent and control childhood obesity among Latino children: the Aventuras Para Ninos Study. Ann Behav Med. 2012/01/05 ed. 2012;43(1):84–100.

16. Jacobson D, Melnyk BM. A primary care healthy choices intervention program for overweight and obese school-age children and their parents. J Pediatr Heal Care [Internet]. 2012/03/01 ed. Elsevier Ltd; 2012 Mar [cited 2014 Nov 18];26(2):126–38. Available from: http://www.ncbi.nlm.nih.gov/pubmed/22360932

17. Schwartz RP, Vitolins MZ, Case LD, Armstrong SC, Perrin EM, Cialone J, et al. The YMCA Healthy, Fit, and Strong Program: a community-based, family-centered, low-cost obesity prevention/treatment pilot study. Child Obes. 2012/11/28 ed. 2012;8(6):577–82.

18. Baker J, Saunders K. Fitter, healthier, happier families: a partnership to treat childhood obesity in the West Midlands. J Public Heal. 2012;126(4):332–4.

19. Bacardí-Gascon M, Pérez-Morales ME, Jiménez-Cruz A, Bacardi-Gascon M, Perez-Morales ME, Jimenez-Cruz A. A six month randomized school intervention and an 18-month follow-up intervention to prevent childhood obesity in Mexican elementary schools. Nutr Hosp [Internet]. 2012/11/02 ed. 2012 [cited 2013 Nov 7];27(3):755–62. Available from: http://www.ncbi.nlm.nih.gov/pubmed/23114940

20. Centis E, Marzocchi R, Di Luzio R, Moscatiello S, Salardi S, Villanova N, et al. A controlled, class-based multicomponent intervention to promote healthy lifestyle and to reduce the burden of childhood obesity. Pediatr Obes [Internet]. 2012/08/23 ed. 2012 Dec [cited 2014 Nov 19];7(6):436–45. Available from: http://www.ncbi.nlm.nih.gov/pubmed/22911919

21. Chen JL, Weiss S, Heyman MB, Lustig RH. Efficacy of a child-centred and family-based program in promoting healthy weight and healthy behaviors in Chinese American children: a randomized controlled study. J Public Heal [Internet]. 2009/11/26 ed. 2010;32(2):219–29. Available from: http://www.ncbi.nlm.nih.gov/pubmed/19933120

22. Chen J-LL, Weiss S, Heyman MB, Cooper B, Lustig RH. The efficacy of the web-based childhood obesity prevention program in Chinese American adolescents (Web ABC study). J Adolesc Heal [Internet]. 2011/07/26 ed. Elsevier Inc.; 2011 Aug [cited 2014 Nov 19];49(2):148–54. Available from: http://www.pubmedcentral.nih.gov/articlerender.fcgi?artid=3143380&tool=pmcentrez&rendertype=abstract

23. Coppins DF, Margetts BM, Fa JL, Brown M, Garrett F, Huelin S. Effectiveness of a multi-disciplinary family-based programme for treating childhood obesity (the Family Project). Eur J Clin Nutr. 2011/04/14 ed. 2011;65(8):903–9.

24. Morgan PJ, Lubans DR, Callister R, Okely a D, Burrows TL, Fletcher R, et al. The “Healthy Dads, Healthy Kids” randomized controlled trial: efficacy of a healthy lifestyle program for overweight fathers and their children. Int J Obes (Lond) [Internet]. 2010/08/11 ed. Nature Publishing Group; 2011 Mar [cited 2013 Oct 22];35(3):436–47. Available from: http://www.ncbi.nlm.nih.gov/pubmed/20697417

25. Morgan PJ, Collins CE, Plotnikoff RC, Callister R, Burrows T, Fletcher R, et al. The `Healthy Dads, Healthy Kids’ community randomized controlled trial: A community-based healthy lifestyle program for fathers and their children. Prev Med (Baltim). 2014 Apr;61:90–9.

26. Duncan S, McPhee JC, Schluter PJ, Zinn C, Smith R, Schofield G. Efficacy of a compulsory homework programme for increasing physical activity and healthy eating in children: the healthy homework pilot study. Int J Behav Nutr Phys Act. 2011/11/17 ed. 2011;8:127.

27. Towey M, Harrell R, Lee B. Evaluation of “one body, one life”: a community-based family intervention for the prevention of obesity in children. J Obes. 2011/10/27 ed. 2011;2011:619643.

28. Burnet DL, Plaut AJ, Wolf SA, Huo D, Solomon MC, Dekayie G, et al. Reach-out: a family-based diabetes prevention program for African American youth. J Natl Med Assoc [Internet]. 2011/06/15 ed. 2011 [cited 2014 Nov 24];103(3):269–77. Available from: http://europepmc.org/abstract/med/21671531

29. Greening L, Harrell KT, Low AK, Fielder CE. Efficacy of a school-based childhood obesity intervention program in a rural southern community: TEAM Mississippi Project. Obes (Silver Spring) [Internet]. 2011;19(6):1213–9. Available from: http://www.ncbi.nlm.nih.gov/pubmed/21233806

30. Golley RK, Magarey AM, Daniels LA. Children’s food and activity patterns following a six-month child weight management program. Int J Pediatr Obes. 2011/08/16 ed. 2011;6(5-6):409–14.

31. Olvera N, Bush J a, Sharma S V, Knox BB, Scherer RL, Butte NF. BOUNCE: a community-based mother-daughter healthy lifestyle intervention for low-income Latino families. Obes (Silver Spring) [Internet]. 2010/01/29 ed. Nature Publishing Group; 2010 Feb [cited 2014 Nov 3];18 Suppl 1(n1s):S102–4. Available from: http://www.ncbi.nlm.nih.gov/pubmed/20107454

32. Olvera N, Scherer R, McLeod J, Graham M, Knox B, Hall K, et al. BOUNCE: an exploratory healthy lifestyle summer intervention for girls. Am J Heal Behav. 2009/10/10 ed. 2010;34(2):144–55.

33. Cliff DP, Okely AD, Morgan PJ, Steele JR, Jones RA, Colyvas K, et al. Movement skills and physical activity in obese children: randomized controlled trial. Med Sci Sport Exerc [Internet]. 2010/05/18 ed. 2011 Jan [cited 2014 Nov 19];43(1):90–100. Available from: http://www.ncbi.nlm.nih.gov/pubmed/20473216

34. Rhodes RE, Naylor P-JJ, McKay HA. Pilot study of a family physical activity planning intervention among parents and their children. J Behav Med [Internet]. 2009/11/26 ed. 2010 Apr [cited 2014 Nov 19];33(2):91–100. Available from: http://www.ncbi.nlm.nih.gov/pubmed/19937106

35. Hardman CAC, Horne PPJ, Lowe CF. A home-based intervention to increase physical activity in girls: The “fit n fun” dudes program. J Exerc Sci Fit [Internet]. 2009 [cited 2014 Nov 24];7(1):1–8. Available from: http://www.sciencedirect.com/science/article/pii/S1728869X09600010

36. Estabrooks PA, Shoup JA, Gattshall M, Dandamudi P, Shetterly S, Xu S. Automated telephone counseling for parents of overweight children: a randomized controlled trial. Am J Prev Med. 2008/12/20 ed. 2009;36(1):35–42.

37. Hovell MF, Nichols JF, Irvin VL, Schmitz KE, Rock CL, Hofstetter CR, et al. Parent/Child training to increase preteens’ calcium, physical activity, and bone density: a controlled trial. Am J Heal Promot [Internet]. 2009/11/26 ed. 2009 [cited 2014 Nov 24];24(2):118–28. Available from: http://www.ncbi.nlm.nih.gov/pubmed/19928484

38. Trost SG, Tang R, Loprinzi PD. Feasibility and efficacy of a church-based intervention to promote physical activity in children. J Phys Act Heal. 2010/01/28 ed. 2009;6(6):741–9.

39. Chen JL, Weiss S, Heyman MB, Vittinghoff E, Lustig R. Pilot study of an individually tailored educational program by mail to promote healthy weight in Chinese American children. J Spec Pediatr Nurs [Internet]. 2008/07/22 ed. 2008 [cited 2014 Nov 24];13(3):212–22. Available from: http://onlinelibrary.wiley.com/doi/10.1111/j.1744-6155.2008.00155.x/full

40. Anand SS, Davis AD, Ahmed R, Jacobs R, Xie C, Hill A, et al. A family-based intervention to promote healthy lifestyles in an aboriginal community in Canada. Can J Public Heal. 2008/12/02 ed. 2007;98(6):447–52.

41. Shelton D, Le Gros K, Norton L, Stanton-Cook S, Morgan J, Masterman P. Randomised controlled trial: A parent-based group education programme for overweight children. J Paediatr Child Heal [Internet]. 2007/09/15 ed. 2007 Dec [cited 2014 Nov 24];43(12):799–805. Available from: http://www.ncbi.nlm.nih.gov/pubmed/17854421

42. Rodearmel SJ, Wyatt HR, Barry MJ, Dong F, Pan D, Israel RG, et al. A family-based approach to preventing excessive weight gain. Obesity (Silver Spring) [Internet]. 2006/09/22 ed. 2006 Aug;14(8):1392–401. Available from: http://www.ncbi.nlm.nih.gov/pubmed/16988082

43. Rooney BL, Gritt LR, Havens SJ, Mathiason MA, Clough EA. Growing healthy families: family use of pedometers to increase physical activity and slow the rate of obesity. WMJ. 2005/09/06 ed. 2005;104(5):54–60.

44. Ransdell LB, Robertson L, Ornes L, Moyer-Mileur L. Generations Exercising Together to Improve Fitness (GET FIT): a pilot study designed to increase physical activity and improve health-related fitness in three generations of women. Women Heal [Internet]. 2005/04/15 ed. 2004 Jan [cited 2014 Nov 27];40(3):77–94. Available from: http://www.ncbi.nlm.nih.gov/pubmed/15829447

45. Beech B, Klesges R. Child-and parent-targeted interventions: the Memphis GEMS pilot study. Ethn Dis [Internet]. 2003 [cited 2014 Nov 24]; Available from: http://www.researchgate.net/publication/10789403_Child-_and_parent-targeted_interventions_the_Memphis_GEMS_pilot_study/file/9c960527162cf2f199.pdf

46. Story M, Sherwood NE, Himes JH, Davis M, Jacobs DR, Cartwright Y, et al. An after-school obesity prevention program for African-American girls: the Minnesota GEMS pilot study. Ethn Dis [Internet]. 2003 Jan [cited 2014 Nov 27];13(1 Suppl 1):S54–64. Available from: http://www.ncbi.nlm.nih.gov/pubmed/12713211

47. Nader PPR, Sallis JJF, Abramson IS, Broyles SL, Patterson TL, Senn K, et al. Family-based cardiovascular risk reduction education among Mexican and Anglo-Americans. Fam Community … [Internet]. 1992 Apr 31 [cited 2014 Nov 24];15(1):57–8. Available from: http://www.researchgate.net/publication/232241059_Family-based_cardiovascular_risk_reduction_education_among_Mexican_and_Anglo-Americans

48. Patterson TL, Sallis JF, Nader PR, Rupp JW, McKenzie TL, Roppe B, et al. Direct observation of physical activity and dietary behaviors in a structured environment: effects of a family-based health promotion program. J Behav Med [Internet]. 1988/10/01 ed. 1988 [cited 2014 Nov 24];11(5):447–58. Available from: http://link.springer.com/article/10.1007/BF00844838

49. Nader PR, Sallis JF, Patterson TL, Abramson IS, Rupp JW, Senn KL, et al. A family approach to cardiovascular risk reduction: results from the San Diego Family Health Project. Health Educ Q [Internet]. 1989/01/01 ed. 1989 Jan [cited 2014 Nov 27];16(2):229–44. Available from: http://www.ncbi.nlm.nih.gov/pubmed/2732065
