# Supplementary material for: Family‐based interventions to increase physical activity in children: a systematic review, meta‐analysis and realist synthesis
Source: Obes Rev. 2016 Jan 12;17(4):345–60. doi: 10.1111/obr.12362 (PMC4819691; doi:10.1111/obr.12362)
Supplement: Supplementary file 2 — Table S2: Results of duplicate quality assessment of studies, using the Effective Public Health Practice Project (EPHPP) Quality Assessment Tool for Quantitative Studies [file OBR-17-345-s002.docx]

*Supplementary* Table 2: Results of duplicate quality assessment of studies, using the Effective Public Health Practice Project (EPHPP) Quality Assessment Tool for Quantitative Studies

| **Study authors**  **and date** | **Intervention name (where available)** | | **Selection bias** | **Study design** | **Confounders** | **Blinding** | **Data collection methods** | **Withdrawal and drop-out** | **Quality assessment rating*** | **Overall score / 12^#^** |
| --- | --- | --- | --- | --- | --- | --- | --- | --- | --- | --- |
| Total studies scoring ‘strong’ per criterion: | | | 8 | 34 | 27 | 4 | 20 | 27 | 3 |  |
|  | |  |  |  |  |  |  |  |  |  |
| Nader 1992 | | The Family Health Project | Strong | Strong | Strong | Strong | Strong | Strong | Strong | 12 |
| Eather 2013 | | Fit4Fun | Strong | Strong | Strong | Moderate | Strong | Strong | Strong | 11 |
| De Bock 2014 | | *None* | Strong | Strong | Strong | Moderate | Strong | Strong | Strong | 11 |
| Morgan 2014 | | Healthy Dads, Healthy Kids | Strong | Strong | Weak | Moderate | Strong | Strong | Moderate | 10 |
| Morrison 2013 | | C-PET | Strong | Strong | Weak | Moderate | Strong | Strong | Moderate | 10 |
| Nyberg 2015 | | The Healthy School Start Study | Weak | Strong | Strong | Moderate | Strong | Moderate | Moderate | 9 |
| Beech 2003 | | GEMS-Memphis | Weak | Strong | Strong | Moderate | Strong | Strong | Moderate | 9 |
| Catenacci 2013 | | AOM | Weak | Moderate | Strong | Strong | Strong | Strong | Moderate | 9 |
| Centis 2012 | | *None* | Strong | Strong | Strong | Weak | Moderate | Strong | Moderate | 9 |
| Chen 2010 | | ABC | Weak | Strong | Strong | Moderate | Strong | Strong | Moderate | 9 |
| Chen 2011 | | ABC | Weak | Strong | Strong | Moderate | Strong | Strong | Moderate | 9 |
| Rooney 2005 | | Growing Healthy Families | Weak | Strong | Strong | Moderate | Strong | Strong | Moderate | 9 |
| Smith 2013 | | MEND 5-7 | Moderate | Moderate | NA | Strong | Weak | Moderate | Moderate | 8 |
| Bacardi-Gascon 2012 | | *None* | Weak | Strong | Strong | Moderate | Moderate | Strong | Moderate | 8 |
| Finkelstein 2013 | | *None* | Weak | Strong | Strong | Moderate | Moderate | Strong | Moderate | 8 |
| Greening 2011 | | TEAM | Strong | Strong | Weak | Moderate | Moderate | Strong | Moderate | 8 |
| Jago 2013 | | Teamplay | Weak | Strong | Strong | Moderate | Strong | Moderate | Moderate | 8 |
| Ransdell 2004 | | GET FIT | Weak | Strong | Moderate | Moderate | Strong | Strong | Moderate | 8 |
| Siwik 2013 | | Choices | Weak | Strong | Strong | Moderate | Moderate | Strong | Moderate | 8 |
| Trost 2009 | | Shining Like Stars | Moderate | Strong | Strong | Moderate | Strong | Weak | Moderate | 8 |
| Newton 2014 | | P-Mobile | Weak | Moderate | Strong | Moderate | Strong | Strong | Moderate | 8 |
| Arredondo 2014 | | *None* | Strong | Moderate | NA | Weak | Weak | Strong | Weak | 7 |
| Anand 2007 | | Share-AP Action | Weak | Strong | Strong | Moderate | Weak | Strong | Weak | 7 |
| Cliff 2010 | | HIKCUPS | Moderate | Strong | Weak | Moderate | Strong | Moderate | Moderate | 7 |
| Duncan 2011 | | Healthy Homework | Weak | Strong | Strong | Moderate | Strong | Weak | Weak | 7 |
| Hovell 2009 | | *None* | Weak | Strong | Strong | Moderate | Weak | Strong | Weak | 7 |
| Nader 1989 | | The Family Health Project | Weak | Strong | Strong | Moderate | Weak | Strong | Weak | 7 |
| Patterson 1988 | | The Family Health Project | Moderate | Strong | Strong | Moderate | Moderate | Weak | Moderate | 7 |
| Story 2003 | | GEMS-Minnesota | Weak | Strong | Strong | Moderate | Strong | Weak | Weak | 7 |
| Estabrooks 2009 | | Family Connections | Weak | Strong | Strong | Weak | Moderate | Moderate | Weak | 6 |
| Golley 2011 | | Triple P | Weak | Strong | Weak | Moderate | Moderate | Strong | Weak | 6 |
| Jacobson 2012 | | Healthy Choices | Weak | Moderate | NA | Strong | Moderate | Strong | Moderate | 6 |
| Olvera 2010 | | BOUNCE | Weak | Moderate | Strong | Weak | Strong | Moderate | Weak | 6 |
| Rhodes 2010 | | *None* | Weak | Strong | Strong | Moderate | Weak | Moderate | Weak | 6 |
| Hardman 2009 | | Fit ‘n’ Fun Dudes | Weak | Strong | Strong | Moderate | Weak | Weak | Weak | 5 |
| Olvera 2010 | | BOUNCE | Weak | Moderate | NA | Weak | Strong | Strong | Weak | 5 |
| Shelton 2007 | | *None* | Weak | Strong | Strong | Moderate | Weak | Weak | Weak | 5 |
| Coppins 2011 | | The Family Project | Weak | Strong | Weak | Moderate | Weak | Moderate | Weak | 4 |
| Crespo 2012 | | The Aventuras Para Ninos Study | Moderate | Strong | Weak | Moderate | Weak | Weak | Weak | 4 |
| Rodearmel 2006 | | *None* | Weak | Strong | Weak | Moderate | Weak | Moderate | Weak | 4 |
| Baker 2012 | | MEND 7-13 | Weak | Moderate | NA | Weak | Weak | Strong | Weak | 3 |
| Burnet 2011 | | Reach Out | Weak | Moderate | NA | Weak | Weak | Strong | Weak | 3 |
| Chen 2008 | | ABC | Weak | Strong | NA | Weak | Weak | Moderate | Weak | 3 |
| Towey 2011 | | One Body One Life | Weak | Moderate | NA | Weak | Weak | Strong | Weak | 3 |
| Barr-Anderson 2013 | | A Family Affair | Weak | Moderate | NA | Weak | Moderate | Weak | Weak | 2 |
| Delamater 2013 | | Fit for Health | Weak | Moderate | NA | Weak | Weak | Moderate | Weak | 2 |
| Schwartz 2012 | | YMCA Healthy, Fit and Strong | Weak | Moderate | NA | Weak | Weak | Moderate | Weak | 2 |

* Studies are rated to give an aggregate overall score of ‘strong’, ‘moderate’ or ‘weak’ (‘strong’ if no ‘weak’ individual-scale ratings are designated, ‘moderate’ if one, and ‘weak’ if two or more).

# Ratings of ‘strong’ are assigned two points, ‘moderate’ one point, and ‘weak’ no points, and then summed to provide additional differentiation between studies.
