# Supplementary material for: Family‐based interventions to increase physical activity in children: a systematic review, meta‐analysis and realist synthesis
Source: Obes Rev. 2016 Jan 12;17(4):345–60. doi: 10.1111/obr.12362 (PMC4819691; doi:10.1111/obr.12362)
Supplement: Supplementary file 3 — File S1: Generic search terms, used in PubMed (title and abstract), Web of Knowledge (topic), Scopus (title, abstract and keywords), Ovid MEDLINE (abstract) and PsycInfo (abstract). [file OBR-17-345-s003.docx]

**Supplementary File 1: Generic search terms, used in PubMed (title and abstract), Web of Knowledge (topic), Scopus (title, abstract and keywords), Ovid MEDLINE (abstract), PsycInfo (abstract).**

**Population:** children OR child OR childhood OR adolescence OR adolescent OR teen OR teenage OR teenager OR teens OR teenagers OR teenaged OR "young people" OR youth OR youths OR boy OR girl OR "school - aged"

AND

**Study design:** intervention OR trial OR randomised OR controlled OR comparison OR experiment OR “quasi-experimental” OR cluster OR programme OR program OR scheme

AND

**Intervention type:** family OR “family-based” OR parent OR mother OR father OR “primary care-giver” OR guardian OR sibling OR brother OR sister OR home OR “home-based”

AND

**Behaviour**: "physical activity" OR "physical activities" OR exercise OR “energy expenditure” OR sport OR sports OR “active travel” OR “sedentary time” OR “sedentary behaviour” OR sitting OR “screen time” OR “television viewing” OR “walking” OR “cycling”

Additionally, the following **MeSH terms** will be used in **PubMed**:

- 1. Child
  2. Adolescent
  3. Family
  4. Physical Activity
  5. Sport
  6. Exercise
  7. Sedentary Lifestyle
  8. Intervention Studies

**The following review articles were also screened for relevant studies:**

Barr-Anderson DJ, Adams-Wynn AW, DiSantis KI, *et al.* Family-focused physical activity, diet and obesity interventions in African-American girls: a systematic review. *Obes Rev* 2013;**14**:29–51.

Salmon J, Booth ML, Phongsavan P, *et al.* Promoting Physical Activity Participation among Children and Adolescents. *Epidemiol Rev* 2007;**29**:144–59.

O’Connor TM, Jago R, Baranowski T. Engaging parents to increase youth physical activity a systematic review. *Am J Prev Med* 2009;**37**:141–9.

van Sluijs EMF, McMinn AM, Griffin SJ. Effectiveness of interventions to promote physical activity in children and adolescents: systematic review of controlled trials. *BMJ* 2007;**335**:703.

van Sluijs EMF, Kriemler S, McMinn AM. The effect of community and family interventions on young people’s physical activity levels: a review of reviews and updated systematic review. *Br J Sport Med* 2011;**45**:914–22.
